# Differential Plasma MicroRNA Profiles in HBeAg Positive and HBeAg Negative Children with Chronic Hepatitis B

**DOI:** 10.1371/journal.pone.0058236

**Published:** 2013-03-04

**Authors:** Thilde Nordmann Winther, Claus Heiner Bang-Berthelsen, Ida Louise Heiberg, Flemming Pociot, Birthe Hogh

**Affiliations:** 1 Department of Paediatrics, Hvidovre Hospital, University of Copenhagen, Copenhagen, Denmark; 2 Diagnostic Unit and Center for Non-Coding RNA in Technology and Health, Glostrup Research Institute, Glostrup Hospital, University of Copenhagen, Glostrup, Denmark; University of Cincinnati College of Medicine, United States of America

## Abstract

**Background and Aim:**

Children chronically infected with hepatitis B virus (HBV) are at high risk of progressive liver disease. However, no treatment is available that is consistently effective in curing chronic hepatitis B (CHB) in children. Improved understanding of the natural course of disease is warranted. Identification of specific microRNA (miRNA) profiles in children chronically infected with HBV may provide insight into the pathogenesis of CHB and lead to advances in the management of children with CHB.

**Patients and Methods:**

MiRNA PCR panels were employed to screen plasma levels of 739 miRNAs in pooled samples from HBeAg positive, HBeAg negative, and healthy children. The three groups’ plasma miRNA profiles were compared, and aberrantly expressed miRNAs were identified. The identified miRNAs were then validated. Individual RT-qPCRs were performed on plasma from 34 HBeAg positive, 26 HBeAg negative, and 60 healthy children.

**Results:**

A panel of 16 plasma miRNAs were identified as aberrantly expressed in HBeAg positive and HBeAg negative children (p<0.001). Levels of all of the miRNAs were upregulated in HBeAg positive children compared with in HBeAg negative children. A positive correlation was furthermore found between plasma levels of the identified miRNAs and HBV DNA (p<0.001).

**Conclusion:**

We are the first to investigate the plasma miRNA profile of children chronically infected with HBV. Our data indicates the existence of a relationship between abundance of circulating miRNAs and immunological stages in the natural course of disease. Certain miRNAs may contribute to the establishment and maintenance of CHB in children. Further studies are warranted to advance understanding of miRNAs in the pathogenesis of CHB, hopefully leading to the identification of future therapeutic targets.

## Introduction

Chronic hepatitis B (CHB) is a global health problem, with more than 350 million people chronically infected worldwide [1]. Infants are at particular risk of developing CHB. When infected perinatally or during early infancy, infection persists in about 90% of infants whereas only 1–5% of patients infected as adults become chronic carriers [2], [3]. CHB in children is associated with a 25% risk of serious adverse outcomes, mainly cirrhosis and hepatocellular carcinoma [4].

HBV is non-cytopathogenic, and liver damage is caused by the host immune system. The natural course of CHB is usually characterised by three stages: The immune tolerant, immune active, and immune inactive stages [5], [6]. Most children are considered to be in the immune tolerant stage, with a high viral load, measurable hepatitis B e antigen (HBeAg), and minimal elevated alanine aminotransferase (ALT). When infection is acquired perinatally or during infancy, the immune tolerant phase can last for 10–30 years. The immune active stage is characterised by a reduction in HBV DNA levels and increased liver damage. The stage of active hepatitis leads to HBeAg seroconversion into anti-HBe antibodies in 2–5% of children annually [7]. HBeAg seroconversion is usually followed by clinical remission and a life-long inactive stage, with low viral load and normal ALT level. Children in the inactive phase have a low risk of liver disease progression, but HBV reactivation can occur and trigger immune mediated liver injury [5], [6].

Improved understanding of the natural course of CHB in children is warranted. It is widely recognised that persistence of HBeAg and level of plasma HBV DNA are associated with risk of cirrhosis and hepatocellular carcinoma [8], [9]. It has furthermore been shown that the amount of circulating HBV DNA plays a key role in disease progression as well as in the transition between immunological stages in the natural course of CHB [10], [11]. However, the exact molecular mechanisms regulating the immunological response are not yet fully understood.

Despite recent advances and developments in CHB treatment strategies, no treatment is available that is consistently effective in curing CHB in children. Anti-HBV therapy currently aims to suppress viral replication, thereby reducing the risk of progressive liver disease, cirrhosis, and hepatocellular carcinoma [12]. It is necessary for new CHB therapies to be developed.

microRNAs (miRNAs) have recently emerged as important posttranscriptional regulators of gene expression with critical functions in health and disease [13]. More than 1500 human miRNAs have been identified to date (miRBase. Available: http://www.mirbase.org. Release 18. Accessed 2012 June 20). Individual miRNAs modulate protein output from hundreds of target genes and may significantly affect gene expression networks [14]. Extra-cellular miRNAs derived from various tissues and organs circulate in the bloodstream and have the capacity to serve as diagnostic markers [15]. miRNAs represent important targets for potential therapeutic agents [13], [16].

It is increasingly clear that miRNAs play a prominent role in the immune system [14], [17]. miRNAs are important in controlling host-viral interactions and regulating viral replication. miRNAs are, moreover, involved in the pathogenesis of chronic infections [18], [19]. Accumulating evidence suggests that miRNAs also modulate HBV replication [20]–[22]. Recently, an aberrant expression of plasma miRNAs was shown in adults with CHB compared with in healthy controls [23]–[25]. To our knowledge, the profile of circulating miRNAs in children with CHB has yet to be investigated.

We hypothesise that miRNAs are important in the immunopathogenesis of HBV infection and specifically that miRNAs contribute to the establishment and maintenance of CHB in children. The study aimed to investigate specific plasma miRNA profiles in children with CHB and to describe the correlation between plasma miRNAs and clinical and virological parameters. We identified a panel of 16 plasma miRNAs as aberrantly expressed in HBeAg positive and HBeAg negative children. All 16 plasma miRNAs levels were upregulated in HBeAg positive children compared with in HBeAg negative children. Additionally, a strong positive correlation was found between the plasma miRNA levels and HBV DNA in children with CHB.

## Patients and Methods

### Patients and Healthy Controls

During the period of July 2005 to April 2011, all children aged 0–18 years who reported to Statens Serum Institut, Copenhagen, Denmark with CHB (n = 202) were invited by letter to participate in the study. Parents of a total of 60 children accepted, and these children were included in the study. Of these children, 34 were HBeAg positive, and 26 were HBeAg negative. CHB is defined as seropositivity for hepatitis B surface antigen (HBsAg) for over six months. The children were followed in accordance with international guidelines [26] and none of them had received any antiviral treatment. Blood samples were obtained at regular clinical visits. Of the 60 children with CHB 25 were followed at the Department of Paediatrics, Hvidovre Hospital, University of Copenhagen, Denmark and 35 were followed at outpatient clinics elsewhere in Denmark. Medical records were obtained and examined for all of the children.

Furthermore, between August 2010 and August 2011, 60 healthy children aged 0–18 years were included as controls. These children were recruited prior to elective surgery for an umbilical or inguinal hernia or prior to orthopaedic surgery at Hvidovre Hospital, University of Copenhagen, Denmark. The children were tested HBsAg negative, and medical records were examined to ensure they were free from known medical conditions. Vaccination against hepatitis B is not included in Denmark’s childhood vaccination programme [27], and none of the children had received vaccination against HBV. Blood samples from seven children were obtained prior to anaesthesia, while blood samples were taken from the majority of children (n = 53) immediately following initiation of anaesthesia (Thiopental).

### Ethical Considerations

The study was performed according to the criteria of the Helsinki II Declaration and was approved by the Ethics Committee Capital Region of Denmark, Reference Number H-KF-255584 and the Danish Data Protection Agency, Journal Number 2009-41-4193. Parents of all participants provided informed written consent prior to any study procedure.

### Blood Samples

Blood samples were collected in EDTA tubes. In Denmark it is standard procedure to process blood samples for plasma isolation within 4 hours of collection. All blood samples collected at Hvidovre Hospital, University of Copenhagen, Denmark were processed according to standard procedure (samples from 25/60 children with CHB and from all the healthy controls). The remaining 35/60 children with CHB were followed at outpatient clinics elsewhere in Denmark. For logistic reasons, it was not possible to process the blood samples for plasma isolation at all of the outpatient clinics. Therefore, to ensure standardised handling all blood samples were sent by mail (at ambient temperature) to the Department of Clinical Biochemistry, Hvidovre Hospital, University of Copenhagen, Denmark for further processing. This implied a processing time of up to 48 hours from collection of blood samples to plasma isolation. Upon arrival at the Department of Clinical Biochemistry, Hvidovre Hospital, University of Copenhagen, Denmark all blood samples were centrifuged at 2500 g for 10 minutes, separated, aliquoted, and stored at −80°C.

### Clinical Chemistry and Virology

Alanine aminotransferase (ALT) from the date of the study-sample collection was obtained from the children’s medical records.

Data on serological status (HBsAg, HBeAg, anti-HBeAg, anti-HAV, anti-HCV, and anti-HIV) was obtained from the children’s medical records.

HBV DNA was quantified using real-time polymerase-chain-reaction (RT-PCR). In brief, total nucleic acids were extracted from 200 µl plasma. The “MagNA Pure 96 DNA and viral NA small volume kit” was used on a MagNA Pure 96 extraction robot. Analyses were performed in accordance with the “Plasma Small Volume Protocol” (Roche, Basel, Switzerland). RT-PCR was performed using 15 µl of extraction per analysis, the QuantiTect Probe PCR kit (Qiagen, Hilden, Germany), 600 nM forward primer HBCPF (5′-ATCTTATCAACACTTCCGGA-3′, nucleotides 2315–2334), 800 nM reverse primer HBCPR (5′-AGATTGAGATCTTCTGCGAC-3′, nucleotides 2434–2415) and 150 nM FAM labelled Tagman Probe HBCPP (5′-FAM-AGGTCCCCTAGAAGAAGAACTCCCT-TAMRA-3′, nucleotides 2360–2384) targeting a 120 bp region (nucleotides 2315–2434) of the core protein gene in hepatitis B virus. The analyses were run on a Stratagene Mx3005p (Agilent Technologies, Santa Clara, California, USA). Cycling conditions were: 2 minutes at 50°C and 15 minutes at 95°C; 40 cycles of 15 seconds at 95°C and 1 minute at 60°C. The quantification was based on an absolute standard curve derived from a HepG2-2.2.15 cell-suspension, which was calibrated using the WHO standard NIBSC (97/750). Over 90% of assays detect a minimum of HBV DNA 50 IU/ml whereas 20% of assays detect HBV DNA 25 IU/ml [28]. The HBV DNA quantification was performed at Statens Serum Institut, Copenhagen, Denmark.

HBV genotyping was performed by direct sequencing of PCR products of the Pre-S region at the Department of Clinical Biochemistry, Aalborg Hospital, Aarhus University, Denmark as previously described [29].

### Design of miRNA Analyses

The miRNA analyses were divided into two phases. Phase One: Screening of aberrantly expressed miRNAs. miRNA polymerase-chain-reaction (PCR) panels containing primers for 739 human miRNAs were employed to screen plasma miRNA levels in samples from HBeAg positive, HBeAg negative, and healthy children. Three samples were analysed: One sample contained plasma from 10 HBeAg positive children, one sample contained plasma from 10 HBeAg negative children, and one sample contained plasma from 10 healthy controls. The three groups’ plasma miRNA profiles were compared, and aberrantly expressed miRNAs were identified. Phase Two: Validation of identified miRNAs. Individual RT-qPCRs were performed on plasma from 34 HBeAg positive, 26 HBeAg negative, and 60 healthy children.

### RNA Extraction

250 µl of plasma were centrifuged at 1,000 g for 5 minutes to remove cell debris. The upper 200 µl were used for RNA extraction. Plasma pools were created by combining 10 samples (200 µl each). Only 200 µl of each plasma pool were used for RNA extraction. Total RNA was extracted from plasma pools/individual plasma samples using the miRNeasy mini kit (Qiagen, Hilden, Germany) per the manufacturer’s instructions with the exception of two modifications: 1.25 µl 0.8 µg/µl MS2 RNA (Roche, Basel, Switzerland) were added to the QIAzol Lysis Reagent, and washing with RPE buffer was repeated x3 instead of x2. RNA concentrations were determined using the NanoDrop 2000c spectrophometer (Thermo Scientific, Waltham, Massachusetts, USA) by measuring absorbance at 260 nm. RNA concentrations ranged from 10.1 to 20.5 ng/µl. Extracted RNA was stored at −80°C.

### cDNA Synthesis

RNAs from both pooled and individual samples were reverse transcribed using the Universal cDNA synthesis kit (Exiqon, Vedbaek, Denmark) per the manufacturer’s instructions. 4 µl total RNA were used for each cDNA synthesis. Reverse transcription was conducted on a GeneAmp PCR System 9700 (Applied Biosystems, Carlsbad, California, USA). cDNA was stored at −20°C prior to use.

### miRNA PCR Panels

miRNA profiles in pooled samples were determined using miRNA PCR panels (human panel I and II V2.M/R), miRCURY LNA™ Universal RT microRNA PCR system (Exiqon, Vedbaek, Denmark). The system consisted of two 384-well PCR plates containing primer sets for one PCR reaction per well. According to the manufacturer the miRNAs covered in the miRNA PCR panels are generally more highly expressed, more likely to be differentially expressed in disease or more often cited in the literature. In total, 739 human miRNAs were analysed together with six reference genes, three inter-plate calibrators, and one control primer set. Thermal conditions were performed on a CFX384 Real-Time thermal cycler (Biorad, Hercules, California, USA) per Exiqon’s instructions. Over 80% of assays detect minimum 100 miRNA copies in the PCR reaction whereas close to 50% detect 10 mRNA copies [30]. All assays were run in duplicate.

### Individual RT-qPCR

The levels of selected miRNAs in individual plasma samples were quantified using individual assays, miRCURY LNA™ Universal RT microRNA PCR system, and specific microRNA LNA™ PCR primer sets (miR-22*, -26a, -99a, -100, -122, -122*, -125b, -192, -192*, -193b, -194, -215, -221, -365, -455-5p, -455-3p, -483-3p, -885-5p, and -1247) (Exiqon, Vedbaek, Denmark). The analyses were performed using a CFX384 Real-Time thermal cycler (Biorad, Hercules, California, USA). This was carried out per Exiqon’s instructions. Individual RT-qPCR was performed in triplicate and included no-template negative controls.

### Identification of Aberrantly Expressed miRNAs

The RT-qPCR results were imported into Microsoft Excel. If not analysed (N/A), the result was replaced with a cycle threshold (C_T_) value of 40 (The RT-qPCR was set to 40 amplification cycles). Three different approaches were used in order to normalise target miRNA expression levels: Global mean; U6; and geometric mean of miR-22*, miR-26a, and miR-221. U6 was selected because it was included as a reference gene in the applied miRNA PCR panel (human panel I and II V2.M/R) and has, furthermore, been used in earlier studies as a reference gene for normalising circulating miRNAs [31], [32]. The combination of miR-22*, miR-26a, and miR-221 has been described as the most stable set of reference genes for RT-qPCR analysis of circulating miRNAs in HBV infected adults [33], [34]. Geometric mean C_T_ values of all miRNAs were calculated for each pool. The relative expression of each miRNA between the three pools was calculated using the comparative C_T_ method [35]. Once normalised with the three strategies, those miRNAs that met the criteria of being a minimum of threefold upregulated or downregulated (when comparing HBeAg positive versus controls, HBeAg negative versus controls, and HBeAg positive versus HBeAg negative) were identified as aberrantly expressed and selected for further analysis.

### Data Analyses of Individual RT-qPCR

Individual RT-qPCR results were imported into Microsoft Excel. If N/A, the results were replaced with a C_T_-value of 40. If the standard deviation of triplicates exceeded one, the sample was reanalysed. The expression levels of target miRNAs were normalised using a combination of miR-22*, miR-26a, and miR-221 and the geometric mean C_T_ values from triplicate wells were calculated. When using volume for normalisation, concordant results were found (data not shown). The comparative C_T_ method was used to analyse the data [35]. The results are shown as –ΔC_T_-values and fold change.

### Pathway Analysis

Pathway analysis was conducted using the DIANA-mirPath Software (http://diana.cslab.ece.ntua.gr/pathways/). Fourteen of the 16 identified miRNAs were combined in one analysis. miR-122* and miR-192* were not included as they were not in the applied database. miRNA targets were predicted using Targetscan 5 (http://www.targetscan.org/). The pathway database used in the analysis was Kyoto Encyclopedia of Genes and Genomes (KEEG) (http://www.genome.jp/kegg/).

### Statistical Analysis

Data was analysed using the Statistical Analysis System Software (SAS), version 9.2 (SAS Institute, Cary, NC, USA). Distributions of continuous data were tested using the Shapiro-Wilk test for normality, and statistical significances were determined using the non-parametric Mann-Whitney test or non-parametric Kruskall-Wallis test. The correlation analyses were performed in two steps, both of which used analysis of variance on ranks (p-values from Chi-Squared tests). First, the different factors were added to the model one at a time (univariate analyses), then all factors were included in the same model (multivariate analyses). Due to multiple testing, p-values <0.004 were regarded as significant (Bonferroni correction).

## Results

### Patient Characteristics

The study included 60 children with CHB and 60 healthy controls. Mean age of children with CHB was 10.1 years (SD 3.9, range 0.9–17.3 years), and mean age of healthy controls was 7.1 years (SD 3.7, range 0.7–15.7 years). The distribution of gender was comparable in both groups (male/female: children with CHB 26/34, healthy controls 33/27). The majority of children with CHB were Asian (62%, n = 37), 17% (n = 10) were African, and 22% (n = 13) were Caucasian, whereas the majority of healthy controls were Caucasian (83%, n = 50), 2% (n = 1) was African, and 15% (n = 9) were Asian. Mean ALT was significantly higher (p<0.0001) in children with CHB than in healthy controls (37.5 U/l versus 14.3 U/l).

Children with CHB were divided into two groups based on HBeAg status: 34 children were HBeAg positive, and 26 children were HBeAg negative. The HBeAg positive children were younger than the HBeAg negative children (mean age 8.8 years versus mean age 12.0 years). This was expected as the relative distribution of HBeAg negative children increases by year. The HBeAg positive children had high HBV DNA levels (mean 5.1E+08), and the HBeAg negative children had low HBV DNA levels (mean 8.5E+02). Furthermore, mean ALT was significantly higher (p<0.0001) in HBeAg positive children than in HBeAg negative children (46.5 U/l versus 25.6 U/l).

HBV genotypes were successfully determined in 49 (82%) of the HBV infected patients; 4 (7%) were genotype A, 13 (22%) were genotype B, 7 (12%) were genotype C, 19 (32%) were genotype D, 5 (8%) were genotype E, and 1 (2%) was genotype F. Among HBeAg positive children genotype A, B, C, D, E, and F were identified and among HBeAg negative children genotype A, B, C, D, and E were identified. Genotyping was not feasible in 11/26 HBeAg negative patients due to HBV DNA levels below the detection limit (HBV DNA <30 IU/ml). Assessment of the genotype distribution between the two groups of children with CHB was therefore not justified. None of the children with CHB showed symptoms of their hepatitis, and none had received antiviral treatment for their hepatitis. All patients were negative for HIV, hepatitis A, and hepatitis C virus.

Patient characteristics are summarised in Table 1.

**Table 1:** Characteristics of children with chronic hepatitis B and of healthy controls.

	Chronic hepatitis B		
	HBeAg pos	HBeAg neg	Healthy controls
**No of patients**	**34**	**26**	**60**
Male	14 (41%)	12 (46%)	33 (55%)
Female	20 (59%)	14 (54%)	27 (45%)
**Age** (years)			
Mean	**8.8**	**12.0**	**7.1**
SD	3.7	3.4	3.7
**Race**			
African	4	6	1
Asian	22	15	9
Caucasian	8	5	50
**ALT** (U/l ref. value 5–45)			
Mean	**46.5**	**25.6**	**14.3**
SD	27.6	12.6	5.3
**HBV DNA** (IU/ml)			
Mean	**5.1E+08**	**8.5E+02**	**NA**
SD	1.2E+09	2.0E+03	
**Genotypes**			
**A**	**2** (6%)	**2** (8%)	
**B**	**11** (32%)	**2** (8%)	
**C**	**5** (15%)	**2** (8%)	
**D**	**12** (35%)	**7** (27%)	
**E**	**3** (9%)	**2** (8%)	
**F**	**1** (3%)	**0** (0%)	
**N/A**	**0** (0%)	**11** (42%)	**60** (100%)

Footnote:

ALT: Alanine aminotransferase.

N/A: Not analysed.

### Aberrantly Expressed miRNAs in Children with CHB

The study aimed to investigate specific plasma miRNA profiles in children with CHB. We initially employed miRNA PCR panels to screen the plasma levels of 739 miRNAs in pooled samples from HBeAg positive, HBeAg negative, and healthy controls. Healthy controls were included primarily to identify CHB-related miRNAs. We successfully measured 287 plasma miRNAs (39% of the tested miRNAs) in HBeAg positive patients, 232 (31%) in HBeAg negative patients, and 254 (34%) in healthy controls (the cut-off level was C_T_ = 35, and all of the miRNAs were measurable in duplicate samples). Residual miRNAs were below the detection limit. Results from the initial screen are shown in Table S1.

The following criteria were defined in order to identify aberrantly expressed miRNAs: miRNA minimum of three fold upregulated or downregulated (when comparing HBeAg positive versus controls, HBeAg negative versus controls, and HBeAg positive versus HBeAg negative). The criteria had to be fulfilled when normalising against global mean; U6; and a combination of miR-22*, miR-26a, and miR-221.

In total, 16 (2%) miRNAs met the criteria above: miR-99a, -100, -122, -122*, -125b, -192, -192*, -193b, -194, -215, -365, -455-5p, -455-3p, -483-3p, -885-5p, and –1247 (Table S2). Of these 16 miRNAs, three pairs of miRNAs were identified: miR-122/−122*, miR-192/−192*, and miR-455-5p/−455-3p. The paired miRNAs are mature and complementary strands respectively, originating from the same hairpin.

### Validation of Aberrantly Expressed miRNAs in Children with Chronic HBV Infection

Next, we validated the identified miRNAs by individual RT-qPCR on plasma from 34 HBeAg positive, 26 HBeAg negative, and 60 healthy children. Ten samples from each group of children were included in both the screening and the validation. No differences were observed in any of the three groups between samples used for “screening and validation” and samples used for “validation only”. Because of this, the results of all of the samples are presented together here.

**Figure 1:**
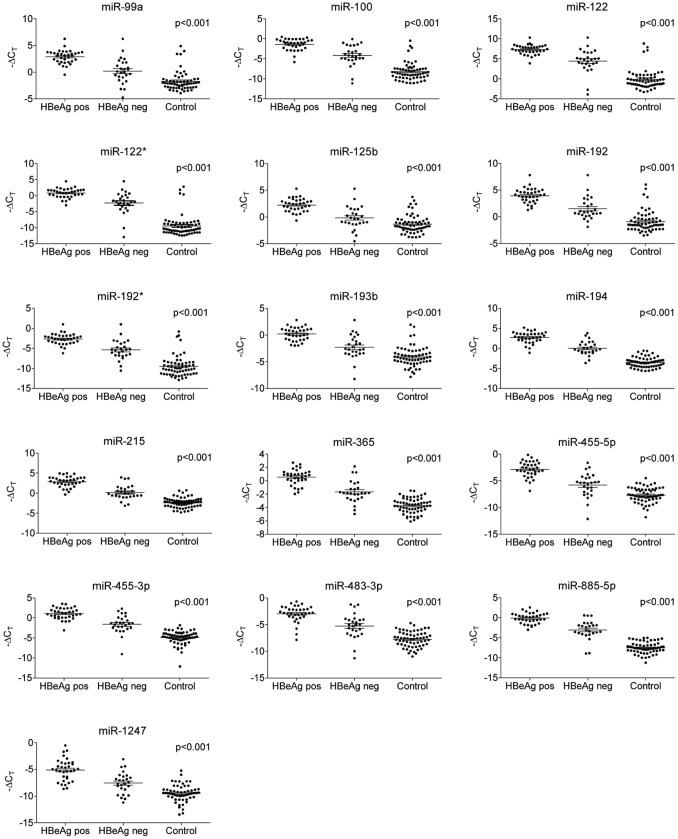
Levels of 16 identified miRNAs in plasma from HBeAg positive, HBeAg negative, and healthy children. A panel of 16 miRNAs was identified as significantly differentially expressed in plasma from 34 HBeAg positive, 26 HBeAg negative, and 60 healthy children. All 16 of the identified miRNAs were highly upregulated in plasma from HBeAg positive children compared with levels in plasma from HBeAg negative children. All miRNAs had their lowest expression in healthy children. Results are corrected for age and gender. –ΔC_T_: −delta cycle threshold. The bars represent geometric means of –ΔC_T_ values ±SEM.

For the 16 identified miRNAs, we compared the miRNA plasma levels in samples from HBeAg positive, HBeAg negative, and healthy children. The miRNAs were consistently differentially expressed between the three groups (p<0.001). Interestingly, levels of all miRNAs turned out to be significantly higher in samples from HBeAg positive children than from HBeAg negative children. All miRNAs had their lowest expression in healthy children. Results are shown in Figure 1.

Plasma levels of circulating miRNAs in HBeAg positive and HBeAg negative children respectively were calculated relative to healthy controls (Table 2).

**Table 2:** Plasma levels of circulating miRNAs in children with CHB relative to healthy controls.

miRNA	Sample	Mean C_T_	SD	Foldchange	SE	P-value*
**99a**	HBeAg pos	26.2	1.4	24	20–30	<0.0001
	HBeAg neg	29.5	1.8	4	3–5	
	Control	30.7	1.2	1		
**100**	HBeAg pos	30.4	2.2	114	88–147	<0.0001
	HBeAg neg	33.9	2.2	16	11–24	
	Control	37.2	1.6	1		
**122**	HBeAg pos	21.8	1.6	171	132–222	<0.0001
	HBeAg neg	25.3	2.5	23	15–37	
	Control	29.1	1.9	1		
**122** *	HBeAg pos	28.3	1.8	1208	859–1697	<0.0001
	HBeAg neg	32.0	2.9	141	81–247	
	Control	38.5	2.6	1		
**125b**	HBeAg pos	26.8	1.5	12	10–15	<0.0001
	HBeAg neg	29.9	1.6	2	2–3	
	Control	30.3	1.1	1		
**192**	HBeAg pos	25.1	1.5	29	23–37	<0.0001
	HBeAg neg	28.2	1.5	5	4–7	
	Control	29.9	1.3	1		
**192** *	HBeAg pos	31.7	1.5	113	85–150	<0.0001
	HBeAg neg	35.1	1.9	17	11–26	
	Control	38.5	1.9	1		
**193b**	HBeAg pos	28.9	1.6	19	15–24	<0.0001
	HBeAg neg	32.0	1.6	3	2–5	
	Control	33.1	1.2	1		
**194**	HBeAg pos	26.6	1.8	75	62–92	<0.0001
	HBeAg neg	29.9	1.3	12	9–15	
	Control	32.7	0.9	1		
**215**	HBeAg pos	26.4	1.7	38	32–46	<0.0001
	HBeAg neg	29.8	1.5	6	4–8	
	Control	31.6	0.9	1		
**365**	HBeAg pos	28.7	1.6	20	17–23	<0.0001
	HBeAg neg	31.6	1.4	4	3–5	
	Control	32.9	0.8	1		
**455-5p**	HBeAg pos	32.2	1.8	28	7–115	<0.0001
	HBeAg neg	35.7	1.8	4	3–5	
	Control	36.8	1.3	1		
**455-3p**	HBeAg pos	28.2	1.7	63	50–79	<0.0001
	HBeAg neg	31.5	2.2	10	7–14	
	Control	34.1	1.5	1		
**483-3p**	HBeAg pos	32.3	2.1	27	22–34	<0.0001
	HBeAg neg	35.2	1.9	6	4–8	
	Control	36.9	0.9	1		
**885-5p**	HBeAg pos	29.4	1.7	168	137–205	<0.0001
	HBeAg neg	33.0	2.0	22	15–30	
	Control	36.7	1.1	1		
**1247**	HBeAg pos	34.4	2.4	20	15–27	<0.0001
	HBeAg neg	37.5	1.7	5	4–7	
	Control	38.6	1.1	1		

Footnote:

* HBeAg positive versus HBeAg negative. HBeAg positive versus controls and HBeAg negative versus controls were all significant at p<0.0001, except for miR-125b HBeAg negative versus controls (p = 0.005) and miR-1247 HBeAg negative versus controls (p = 0.002).

Blood samples from 35/60 children with CHB were not processed according to standard procedure. The samples were collected in EDTA tubes and immediately hereafter sent by mail to the Department of Clinical Biochemistry, Hvidovre Hospital, University of Copenhagen, Denmark for further processing. The freight implied a processing time of up to 48 hours from collection of blood samples to plasma isolation. To assess the impact of extended processing time on the plasma miRNA profile we compared the plasma levels of 16 miRNAs in 25 samples (16 HBeAg positive and 9 HBeAg negative) processed as generally recommended (within 4 hours of collection) and in 35 samples (18 HBeAg positive and 17 HBeAg negative) processed after a delay of up to 48 hours. No difference was found (Table S3).

The majority (53/60) of healthy controls submitted a blood sample immediately after initiation of anaesthesia (Thiopental). To investigate if the anaesthetic agents affected plasma miRNA levels, we compared the plasma levels of all 16 miRNAs in blood samples obtained before and after anaesthesia respectively. No difference was found (data not shown).

### Correlation between Circulating miRNAs and Clinical and Virological Parameters

The HBeAg positive children were younger than the HBeAg negative children. We thus analysed the association between levels of circulating miRNAs and ages of children at sample date. The levels of miR-100, -122, and -122* were shown to correlate with age (p<0.001). However, this relationship was not confirmed when adjusted for HBeAg status (p = 0.06, p = 0.007, and p = 0.06, respectively (Due to multiple testing only p<0.004 was regarded as statistically significant)).

No correlation was found between plasma miRNA levels and gender or race (data not shown).

**Figure 2:**
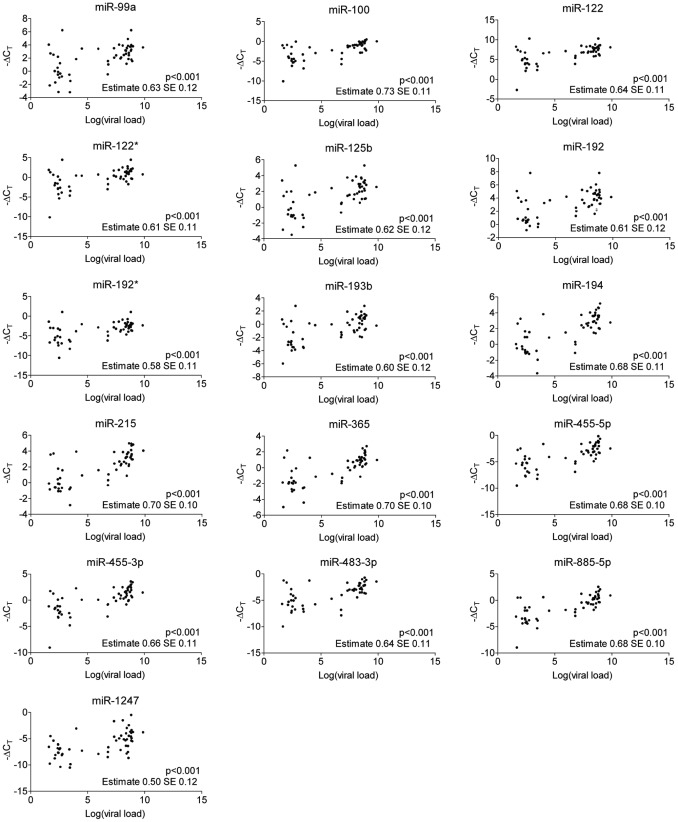
Correlation between circulating miRNAs and HBV DNA. The relationship between circulating miRNAs and viral load was investigated and a strong positive correlation was found between plasma levels of all 16 miRNAs and HBV DNA. Results are corrected for age, gender, and ALT.

We also investigated the relationship between circulating miRNAs and viral load and found a very strong positive correlation between plasma levels of all 16 miRNAs and HBV DNA (p<0.001). This correlation persisted when adjusted for age, gender, and ALT. Healthy controls were excluded from these analyses. Results are shown in Figure 2.

ALT is a marker for liver damage. We looked for an association between plasma ALT and levels of circulating miRNAs in children with CHB. Surprisingly, no such correlation was observed. When correlating the quantity of plasma miRNAs in HBeAg positive and HBeAg negative children with ALT, the results were insignificant (p value between 0.3 and 0.9) (data not shown). However, when including ALT from HBeAg positive, HBeAg negative, and healthy controls in the analyses, we found a significant correlation between the 16 miRNAs and ALT (p<0.001) (data not shown).

There was no correlation between circulating miRNAs and genotype. Because genotyping was only feasible for 15 out of 26 HBeAg negative children, genotype was not included in the multivariate analyses.

### Gene Set Enrichment Analysis

A gene set enrichment analysis was performed to gain insight into the biological functions of the miRNAs differentially expressed in HBeAg positive and HBeAg negative children. Fourteen of the 16 identified miRNAs were combined in the analysis (miR-122* and miR-192* were excluded). The ten pathways identified with highest significance (p<0.0002) were all signalling pathways or cancer-specific pathways (Table 3).

**Table 3:** Gene set enrichment analysis.

Pathway name	Kegg ID	Genes	P-value
Insulin signaling pathway	hsa04910	33	4.5E-10
Colorectal cancer	hsa05210	21	7.3E-07
Acute myeloid leukemia	hsa05221	15	6.7E-06
Prostate cancer	hsa05215	20	1.1E-05
Glioma	hsa05214	16	1.5E-05
MAPK signaling pathway	hsa04010	41	1.5E-05
ErbB signaling pathway	hsa04012	19	4.8E-05
Chronic myeloid leukemia	hsa05220	17	8.1E-05
Wnt signaling pathway	hsa04310	26	1.4E-04
Melanoma	hsa05218	16	1.6E-04

Footnote: Fourteen of the 16 identified miRNAs were combined in a gene set enrichment analysis. miR-122* and miR-192* were not included as they were not in the applied database. The ten pathways identified with highest significance are shown.

## Discussion

Infants are at particular risk of developing CHB. When infected perinatally or during early infancy, infection persists in about 90% of infants whereas only 1–5% of patients infected as adults become chronic carriers [2], [3]. Moreover, children chronically infected with HBV have a 25% risk of progressive liver disease, liver cirrhosis, and hepatocellular carcinoma [4]. No treatment is available that is consistently effective in curing CHB in children [11]. Identification of specific miRNA profiles in children chronically infected with HBV and an improved understanding of the role played by miRNAs in the pathogenesis of CHB may lead to advances in the management of children with CHB.

We are the first to investigate the plasma miRNA profile of children with CHB. A panel of 16 miRNAs was identified as significantly differentially expressed in plasma from HBeAg positive, HBeAg negative, and healthy children. The miRNAs were: miR-99a, -100, -122, -122*, -125b, -192, -192*, -193b, -194, -215, -365, -455-5p, -455-3p, -483-3p, -885-5p, and -1247. Interestingly, with the exception of miR-1247, all of the miRNAs have previously been shown to associate with CHB in adults [25], [36], [37], substantiating the importance of this unique panel of miRNAs in the pathogenesis of CHB in children. miR-122 is the most abundant liver-specific miRNA and was recently identified as a sensitive, though not specific, marker for liver injury [38]. As far as HBV is concerned, miR-122 was shown to inhibit viral replication [37]. Although the remainder of the identified miRNAs in this study are described in relation to the liver and/or the immune system (http://www.microrna.org) [39], the biological functions of the miRNAs remain largely unclear. The strategy of broad miRNA profiling may reveal an etiology-specific miRNA signature.

The differentiation between the presence and absence of HBeAg is of great importance for children with CHB. We show that all 16 of the identified miRNAs were highly upregulated in plasma from HBeAg positive children compared with levels in plasma from HBeAg negative children. No previous studies have identified a whole panel of miRNAs differentially expressed in HBeAg positive and HBeAg negative patients. However, one study compared the levels of nine miRNAs in serum from seven HBeAg positive and nine HBeAg negative adults and showed an association between presence of HBeAg and level of miR-122 as well as level of miR-194 [37].

Interestingly, blood samples obtained from one child before and after HBeAg seroconversion to anti-HBe antibodies showed a significant decrease in plasma levels of the 16 identified miRNAs in the present study after seroconversion (TNW unpublished data). Although it would be unjustified to draw conclusions from observations on a single child, this does further suggest a role for certain miRNAs in the pathogenesis of CHB in children.

Quantity of plasma HBV DNA is key to progression of CHB as well as to transition between immunological stages [10]. Our study investigated the relationship between circulating miRNAs and viral load and found a strong positive correlation between plasma levels of all 16 miRNAs and HBV DNA. These results suggest that miRNAs may play a role in viral replication. Inhibition of HBV replication by miR-122 has been documented [37], and in agreement with our results a recent study demonstrated a correlation between serum levels of miR-122 and HBV DNA in samples from adults with CHB [23].

Our data indicates the existence of a relationship between abundance of circulating miRNAs and immunological stages in the natural course of CHB. In line with this, we speculate that miRNAs are involved in the pathogenesis of HBV infection and specifically that certain miRNAs contribute to the establishment and maintenance of CHB in children.

The extent of liver damage varies in CHB’s different immunological stages [5], [6]. Current clinical practice utilises ALT as a marker for liver damage. In the present study, HBeAg positive children had a mean ALT just above the normal range, indicating a low grade of liver inflammation. HBeAg negative children had a mean ALT within the normal range. However, although the ALT was within normal range, it was still significantly higher than the mean ALT in healthy controls, suggesting some inflammatory activity even in the children regarded as immune inactive.

miR-122 has recently emerged as a sensitive marker for liver damage [38]. Surprisingly, this study did not find an association between levels of circulating miRNAs and ALT. It is tempting to suggest that miRNAs are more sensitive markers for liver injury than ALT and, as a consequence, that the levels of circulating miRNAs increase at minimal liver inflammation even when ALT remains within normal range.

It is widely recognised that miRNAs are transcribed in cells but present in the bloodstream [15]. We argue that increased plasma miRNA levels in children with CHB are related to the host’s immune response and subsequent liver pathology. miRNAs may passively leak into the plasma from damaged or dying cells as a result of apoptosis or necrosis.

Another explanation for the increased level of circulating miRNAs in children with CHB is that cells actively secrete miRNAs in order to regulate other cell types and tissues [40], [41]. We speculate that infected hepatocytes secrete miRNAs to attract immune cells and thereby activate the immune system or perhaps that HBV triggers the release of miRNAs in order to attain optimal conditions for virus replication.

A dysregulation of miRNAs in children with CHB is supported by the fact that three out of 16 identified miRNAs in this study were complementary strands (miR-122*, miR-192*, and miR-455-3p). Studies investigating miRNA biogenesis have shown that complementary miRNA strands are degraded as soon as they are cleaved from their mature miRNA strands [14]. Measurable levels of complementary miRNA strands may therefore indicate cellular disruption and/or miRNA dysregulation, which could be caused by HBV.

As children with CHB are at risk of severe adverse outcomes, life-long follow-up is warranted. Treatment is recommended if any signs of liver damage are detected [12], [26]. Current anti-HBV therapy aims to suppress viral replication and to achieve durable HBeAg seroconversion. Unfortunately, none of the existing treatments are consistently effective in curing CHB in children [11]. miRNAs have shown great potential as targets for new therapeutic agents [13], [42]. It was recently reported, for example, that miR-122 silencing in chimpanzees with chronic hepatitis C led to potent and sustained inhibition of hepatitis C virus replication [16], [43]. In the present study, a gene set enrichment analysis was performed on a combination of the miRNAs differentially expressed in HBeAg positive and HBeAg negative children (except for miR-122* and miR-192*). Interestingly, the ten pathways identified with highest significance (p<0.0002) were all signalling pathways or cancer specific pathways (Table 3). Broad miRNA profiling may thus provide insight into the pathogeneses of CHB in children and lead to targets for the development of new treatment strategies.

In conclusion, we are the first to investigate the miRNA profile of children chronically infected with HBV. A panel of 16 plasma miRNAs was identified as aberrantly expressed in HBeAg positive and HBeAg negative children. Levels of all 16 plasma miRNAs were upregulated in HBeAg positive children compared with in HBeAg negative children. A strong positive correlation was found between plasma levels of the identified miRNAs and HBV DNA. Our data indicates the existence of a relationship between abundance of circulating miRNAs and immunological stages in the natural course of disease. Certain miRNAs may contribute to the establishment and maintenance of CHB in children. Further studies are warranted to advance understanding of the role played by miRNAs in the pathogenesis of CHB, hopefully leading to the identification of future therapeutic targets.

## Supporting Information

Table S1 **Data from initial screen of miRNA levels in pooled plasma samples from HBeAg positive, HBeAg negative, and healthy controls. miRNA PCR panels from Exiqon were applied and results of human panel I and II V2.M/R are shown in Table A and B, respectively.**
(DOC)

Table S2 **miRNAs identified as aberrantly expressed in the screening phase using three different normalisation strategies, reported as fold change.**
(DOC)

Table S3 **Plasma levels of circulating miRNAs and varying time from collection of blood samples to centrifugation and separation.**
(DOC)
