# Supplementary material for: Differential Plasma MicroRNA Profiles in HBeAg Positive and HBeAg Negative Children with Chronic Hepatitis B
Source: PLoS One. 2013 Mar 4;8(3):e58236. doi: 10.1371/journal.pone.0058236 (PMC3587589; doi:10.1371/journal.pone.0058236)
Supplement: Table S1 — Data from initial screen of miRNA levels in pooled plasma samples from HBeAg positive, HBeAg negative, and healthy controls. miRNA PCR panels from Exiqon were applied and results of human panel I and II V2.M/R are shown in Table A and B, respectively. (DOC) [file pone.0058236.s001.doc]

Table S1, Data from initial screen of miRNA levels in pooled plasma samples from HBeAg positive, HBeAg negative, and healthy controls. miRNA PCR panels from Exiqon were applied and results of human panel I and II V2.M/R are shown in Table A and B, respectively.

A,

|  | Human panel **I** V2.M/R, raw C_T_ values | | | | | |
| --- | --- | --- | --- | --- | --- | --- |
|  | **HBeAg positive pool** | | **HBeAg negative pool** | | **Control pool** | |
| **miRNA** | Replicate 1 | Replicate 2 | Replicate 1 | Replicate 2 | Replicate 1 | Replicate 2 |
| hsa-miR-379 | 36.72 | 39.09 |  | 38.44 | 35.96 | 36.49 |
| hsa-miR-217 | 34.78 | 37.09 |  |  |  |  |
| hsa-miR-337-5p | 39.19 | 35.97 |  | 36.59 | 37.23 | 33.58 |
| hsa-miR-328 | 29.61 | 29.97 | 30.76 | 30.48 | 30.23 | 29.75 |
| hsa-miR-374b* | 36.94 | 39.53 |  |  |  | 37.40 |
| hsa-miR-143 | 30.28 | 29.02 | 31.19 | 28.82 | 30.47 | 28.36 |
| hsa-miR-623 |  |  |  | 39.06 |  | 38.97 |
| hsa-miR-520c-3p | 37.62 |  |  |  | 37.29 |  |
| hsa-miR-557 |  |  |  |  |  |  |
| hsa-miR-218 | 38.09 | 36.17 | 35.13 |  | 36.05 | 36.51 |
| hsa-miR-136 | 35.63 | 31.02 | 35.28 | 31.50 | 33.38 | 29.81 |
| hsa-miR-127-5p |  | 37.22 |  | 37.09 |  | 36.19 |
| hsa-miR-140-5p | 30.57 | 31.21 | 31.15 | 31.50 | 30.36 | 30.12 |
| hsa-miR-31* |  | 37.59 |  | 38.96 | 37.35 | 37.05 |
| hsa-miR-20b* | 31.50 | 32.96 | 33.87 | 35.42 | 34.06 | 35.32 |
| hsa-miR-325 |  |  |  |  |  |  |
| hsa-miR-509-3-5p |  |  |  |  |  |  |
| hsa-miR-210 | 32.27 | 30.57 | 32.84 | 31.70 | 32.63 | 30.63 |
| hsa-miR-199b-5p | 39.24 | 35.06 | 38.17 | 34.38 | 37.44 | 33.02 |
| hsa-miR-194 | 25.54 | 25.87 | 29.45 | 28.98 | 34.22 | 32.35 |
| hsa-let-7g | 27.00 | 27.04 | 28.36 | 27.77 | 27.71 | 26.66 |
| hsa-miR-203 | 35.53 | 35.16 | 39.06 | 39.22 |  |  |
| hsa-miR-181a* | 36.56 | 34.24 | 36.63 | 37.78 | 35.98 | 35.53 |
| hsa-miR-934 | 34.64 | 34.13 | 34.00 | 34.52 | 34.14 | 34.53 |
| hsa-miR-551b | 33.02 | 32.54 | 34.63 | 32.70 | 33.27 | 32.12 |
| UniSp6 CP | 12.50 | 29.28 | 13.05 | 28.36 | 12.65 | 28.55 |
| hsa-miR-524-3p |  | 38.33 |  |  |  |  |
| hsa-miR-7 | 34.31 | 36.18 | 34.45 | 35.36 | 34.85 | 37.36 |
| hsa-miR-486-5p | 26.01 | 27.32 | 26.27 | 27.22 | 25.10 | 25.43 |
| hsa-miR-30c | 24.54 | 25.05 | 27.59 | 27.83 | 28.26 | 28.26 |
| hsa-miR-301b | 33.90 | 34.32 | 34.43 | 34.02 | 33.66 | 32.85 |
| hsa-miR-128 | 33.13 | 30.50 | 33.49 | 31.21 | 33.59 | 30.51 |
| hsa-miR-329 | 35.54 | 33.54 | 36.09 | 33.80 | 34.20 | 33.02 |
| hsa-miR-224 | 37.95 | 34.89 | 38.54 | 35.37 | 38.85 | 35.05 |
| hsa-miR-487b | 36.81 | 33.72 | 35.70 | 34.44 | 35.08 | 32.66 |
| hsa-miR-130a | 30.85 | 27.51 | 31.41 | 28.03 | 31.19 | 27.58 |
| hsa-miR-138 |  | 38.15 | 37.22 | 37.21 | 38.08 | 36.93 |
| hsa-miR-26a-2* | 36.78 | 38.71 |  | 39.76 | 39.78 | 38.28 |
| hsa-miR-378 | 26.88 | 26.86 | 29.85 | 29.50 | 30.42 | 30.09 |
| hsa-miR-381 | 37.14 | 34.83 |  | 34.35 | 37.99 | 33.39 |
| hsa-miR-671-5p | 36.68 | 34.38 | 38.50 | 34.15 | 35.75 | 33.71 |
| hsa-miR-521 |  |  |  |  |  | 39.49 |
| hsa-miR-221 | 27.49 | 26.41 | 28.05 | 26.60 | 27.25 | 25.38 |
| hsa-miR-142-5p | 29.61 | 27.54 | 30.46 | 27.54 | 29.77 | 26.62 |
| hsa-miR-132 | 32.18 | 31.46 | 32.56 | 31.26 | 32.73 | 30.72 |
| hsa-miR-424 | 30.00 | 28.32 | 30.59 | 28.63 | 29.69 | 27.55 |
| UniSp3 IPC | 22.97 | 22.67 | 22.36 | 22.44 | 22.67 | 22.17 |
| hsa-miR-374a | 31.33 | 32.17 | 33.12 | 33.76 | 33.86 | 31.85 |
| hsa-miR-532-5p | 32.10 | 33.10 | 33.30 | 32.52 | 31.92 | 31.84 |
| hsa-miR-99a | 25.34 | 26.03 | 28.82 | 28.81 | 31.98 | 31.35 |
| hsa-miR-92a-1* | 34.92 | 35.45 |  | 37.75 |  | 37.29 |
| hsa-miR-125b | 25.17 | 26.11 | 28.58 | 29.02 | 30.51 | 30.63 |
| hsa-miR-185 | 26.49 | 27.46 | 26.99 | 27.07 | 26.32 | 26.25 |
| hsa-miR-25 | 27.04 | 27.65 | 27.81 | 27.88 | 26.86 | 26.92 |
| hsa-miR-524-5p | 37.35 | 38.20 |  |  |  | 38.22 |
| hsa-miR-20a | 24.26 | 24.80 | 24.85 | 25.41 | 24.21 | 24.50 |
| hsa-miR-765 |  | 37.77 |  | 38.26 |  | 37.53 |
| hsa-miR-24 | 24.50 | 24.86 | 25.18 | 25.52 | 24.92 | 24.85 |
| hsa-miR-369-5p | 35.06 | 35.52 | 35.41 | 36.05 | 34.08 | 34.01 |
| hsa-miR-425 | 29.01 | 28.59 | 29.18 | 29.01 | 28.73 | 28.22 |
| hsa-miR-590-5p | 28.70 | 29.80 | 29.08 | 30.14 | 28.67 | 29.76 |
| hsa-miR-760 | 34.57 | 35.03 | 34.19 | 34.68 | 35.69 | 35.01 |
| hsa-miR-574-3p | 27.44 | 28.32 | 29.99 | 31.02 | 30.16 | 30.67 |
| hsa-miR-130b | 33.88 | 31.98 | 34.48 | 32.26 | 35.03 | 31.37 |
| hsa-let-7e | 32.04 | 32.80 | 33.13 | 33.12 | 32.43 | 32.34 |
| hsa-miR-133b | 34.51 | 32.56 | 33.71 | 32.58 | 33.77 | 32.12 |
| hsa-miR-542-5p |  | 35.19 | 36.78 | 37.52 | 37.19 | 34.11 |
| hsa-miR-23a | 25.19 | 25.65 | 25.93 | 25.60 | 24.98 | 24.72 |
| hsa-miR-193b | 28.40 | 27.52 | 31.72 | 30.61 | 33.90 | 32.16 |
| UniSp3 IPC | 23.22 | 22.59 | 23.66 | 22.63 | 23.10 | 22.27 |
| hsa-miR-518c* |  | 39.79 |  | 38.75 |  | 37.78 |
| hsa-miR-204 | 31.44 | 31.44 | 36.11 | 35.20 | 36.29 | 36.04 |
| hsa-miR-933 | 38.03 | 39.11 | 38.83 | 36.70 |  |  |
| SNORD49A | 37.23 |  |  |  | 37.80 |  |
| hsa-miR-452 | 33.95 | 34.49 | 34.23 | 35.27 | 34.43 | 35.32 |
| hsa-miR-215 | 24.82 | 24.82 | 28.58 | 28.33 | 30.86 | 31.34 |
| hsa-miR-141 | 33.07 | 34.08 | 34.59 | 34.82 | 33.15 | 33.15 |
| hsa-miR-374b | 29.30 | 30.11 | 30.80 | 31.39 | 29.49 | 30.25 |
| hsa-miR-668 | 36.77 | 39.41 | 36.62 |  | 37.04 | 36.17 |
| hsa-miR-33a | 33.26 | 28.38 | 34.30 | 28.86 | 33.71 | 28.08 |
| hsa-miR-101 | 24.23 | 24.72 | 26.24 | 26.20 | 25.40 | 25.76 |
| hsa-miR-30c-2* |  | 34.90 |  | 35.60 |  | 36.40 |
| hsa-miR-331-3p | 29.59 | 28.96 | 31.56 | 30.43 | 30.78 | 29.57 |
| hsa-miR-340 | 33.58 | 32.43 | 33.86 | 33.21 | 34.60 | 33.62 |
| hsa-miR-196a | 38.70 | 37.86 |  | 39.65 | 38.81 | 37.27 |
| hsa-miR-888 |  | 37.07 | 38.11 | 37.71 | 38.33 | 37.96 |
| hsa-miR-330-3p | 34.30 | 35.41 | 35.01 | 34.09 | 34.27 | 33.36 |
| hsa-miR-570 | 36.64 | 34.45 | 37.80 | 36.69 | 37.01 | 35.01 |
| hsa-miR-518c |  | 39.34 |  | 36.18 |  | 36.53 |
| hsa-miR-200a | 33.46 | 33.68 | 35.86 | 36.07 | 35.37 | 35.50 |
| hsa-miR-188-5p | 38.95 | 34.18 | 36.56 | 34.50 | 38.62 | 33.72 |
| hsa-miR-26a | 25.58 | 25.66 | 27.23 | 27.11 | 26.40 | 26.11 |
| hsa-miR-99b | 30.88 | 31.47 | 31.34 | 31.41 | 31.01 | 30.49 |
| hsa-miR-431 | 35.28 | 34.41 | 36.02 | 33.88 | 34.76 | 32.43 |
| hsa-miR-23b | 24.81 | 25.09 | 27.10 | 26.89 | 27.13 | 26.49 |
| hsa-miR-367 |  |  |  |  |  |  |
| hsa-miR-505 | 29.57 | 30.39 | 31.89 | 32.09 | 32.28 | 32.49 |
| hsa-miR-18a | 29.31 | 30.31 | 29.89 | 30.29 | 29.13 | 29.15 |
| hsa-miR-92a | 24.07 | 24.48 | 24.83 | 25.04 | 24.33 | 24.09 |
| hsa-miR-500a | 33.99 | 34.05 | 36.44 | 37.12 | 35.51 | 34.43 |
| hsa-miR-887 | 39.38 | 35.98 | 38.05 | 35.58 |  | 35.91 |
| hsa-miR-491-3p | 38.29 | 37.97 |  | 38.19 | 38.07 | 38.30 |
| hsa-miR-423-3p | 28.03 | 27.41 | 28.76 | 28.07 | 28.54 | 27.32 |
| hsa-miR-126 | 24.13 | 25.04 | 25.01 | 25.21 | 23.98 | 23.90 |
| hsa-miR-622 | 35.14 | 35.49 | 36.43 | 35.87 | 35.89 | 35.02 |
| hsa-miR-376b | 34.70 | 31.94 | 35.21 | 32.76 | 33.30 | 31.09 |
| hsa-miR-302c |  |  |  |  |  |  |
| hsa-miR-185* | 37.27 | 34.33 | 38.60 | 34.52 | 35.99 | 34.42 |
| hsa-miR-339-5p | 31.40 | 31.37 | 32.67 | 32.72 | 32.22 | 31.53 |
| hsa-miR-873 |  | 39.05 |  |  |  | 36.53 |
| hsa-miR-323-3p | 34.72 | 34.34 | 35.53 | 34.79 | 33.67 | 33.24 |
| hsa-miR-181d | 35.05 | 35.37 | 36.59 | 34.94 | 35.75 | 33.24 |
| hsa-miR-125a-5p | 30.27 | 31.78 | 30.88 | 31.57 | 30.04 | 30.13 |
| hsa-miR-129-5p |  | 35.97 |  | 37.80 | 39.02 | 36.40 |
| hsa-miR-492 |  |  |  |  |  |  |
| hsa-miR-519d |  |  |  | 38.03 |  |  |
| UniSp3 IPC | 22.12 | 21.97 | 22.15 | 22.16 | 23.23 | 21.74 |
| hsa-miR-302d |  | 38.64 |  |  |  |  |
| hsa-miR-346 | 33.63 | 35.22 | 34.02 | 35.13 | 35.07 | 34.20 |
| hsa-miR-151-3p | 28.57 | 28.85 | 29.39 | 29.84 | 30.04 | 28.58 |
| hsa-miR-493 | 35.65 | 35.51 | 34.64 | 35.43 | 33.98 | 33.60 |
| hsa-miR-423-5p | 29.18 | 28.24 | 30.26 | 28.54 | 29.96 | 28.02 |
| hsa-miR-99a* | 32.56 | 31.93 | 35.70 | 34.45 | 35.69 | 36.24 |
| hsa-miR-10a | 34.46 | 34.82 | 34.32 | 34.54 | 34.87 | 35.30 |
| hsa-miR-202 |  | 39.46 |  |  |  | 37.76 |
| hsa-miR-10b | 33.32 | 34.35 | 34.53 | 34.11 | 34.08 | 33.75 |
| hsa-miR-503 | 36.25 | 32.34 | 35.93 | 32.82 |  | 31.85 |
| hsa-miR-890 |  |  |  |  |  |  |
| hsa-miR-30d | 27.31 | 28.12 | 29.53 | 29.33 | 28.35 | 29.00 |
| hsa-miR-514 |  |  |  |  |  |  |
| hsa-miR-16 | 23.25 | 23.34 | 23.66 | 23.47 | 22.53 | 22.12 |
| hsa-miR-150 | 26.97 | 27.89 | 27.53 | 27.97 | 27.04 | 27.19 |
| hsa-miR-654-5p | 35.32 | 33.08 | 37.24 | 33.02 | 37.12 | 32.39 |
| hsa-miR-545 | 37.95 | 34.01 | 37.59 | 35.33 | 37.70 | 34.72 |
| hsa-miR-29b-2* | 34.50 | 33.59 | 36.01 | 34.64 | 37.04 | 33.83 |
| hsa-miR-491-5p | 33.85 | 34.14 | 34.93 | 35.25 | 33.81 | 33.45 |
| hsa-miR-92b | 33.50 | 34.21 | 34.97 | 34.32 | 34.28 | 33.33 |
| hsa-miR-665 | 36.94 | 33.23 | 39.02 | 33.49 |  | 32.82 |
| hsa-miR-506 |  |  |  | 39.83 |  |  |
| hsa-miR-363 | 32.54 | 31.44 | 32.96 | 30.99 | 31.67 | 29.88 |
| hsa-miR-663 | 38.98 | 33.80 | 36.09 | 33.38 | 38.11 | 33.65 |
| hsa-miR-651 | 35.46 | 33.30 | 36.36 | 34.89 | 35.30 | 33.16 |
| hsa-miR-342-3p | 28.23 | 28.93 | 29.36 | 29.38 | 28.78 | 28.51 |
| hsa-miR-432 | 34.30 | 35.08 | 35.79 | 35.68 | 35.18 | 32.88 |
| hsa-miR-154* |  | 38.61 |  | 37.21 | 39.64 | 35.79 |
| hsa-miR-27a | 25.06 | 25.74 | 25.60 | 25.84 | 25.36 | 25.38 |
| hsa-miR-376c | 29.45 | 30.07 | 30.26 | 30.33 | 29.08 | 28.86 |
| hsa-miR-940 | 35.81 | 33.17 | 36.60 | 33.90 | 35.19 | 33.57 |
| hsa-miR-22* | 32.23 | 29.64 | 33.21 | 30.53 | 33.20 | 29.57 |
| hsa-miR-34c-5p |  |  |  | 39.39 | 37.50 | 37.15 |
| hsa-miR-885-5p | 28.16 | 27.97 | 32.18 | 31.56 | 36.22 | 36.00 |
| hsa-miR-320a | 24.92 | 25.20 | 26.09 | 26.06 | 26.22 | 25.17 |
| hsa-miR-18b | 28.21 | 29.15 | 29.11 | 29.29 | 28.04 | 28.35 |
| hsa-miR-187 | 35.44 |  |  |  | 37.83 |  |
| hsa-miR-516b |  |  |  |  |  |  |
| hsa-miR-302c* |  |  |  | 39.22 |  |  |
| hsa-miR-548b-3p | 37.68 | 33.54 | 39.37 | 34.32 |  | 38.17 |
| hsa-miR-186 | 28.95 | 28.64 | 30.10 | 29.48 | 29.25 | 28.41 |
| hsa-miR-199a-5p | 30.81 | 27.36 | 31.28 | 27.34 | 30.36 | 26.54 |
| hsa-miR-155 | 33.87 | 33.06 | 33.67 | 33.46 | 33.25 | 32.61 |
| hsa-miR-107 | 28.90 | 27.04 | 29.69 | 27.73 | 29.16 | 26.56 |
| hsa-miR-302b |  |  |  |  |  |  |
| hsa-miR-662 |  | 36.94 |  | 36.75 |  | 36.38 |
| hsa-miR-30a | 30.13 | 30.86 | 33.60 | 32.64 | 33.90 | 32.77 |
| hsa-miR-302d* |  | 37.10 | 39.44 | 36.37 |  | 36.91 |
| hsa-miR-484 | 27.89 | 27.89 | 28.80 | 28.18 | 28.22 | 27.24 |
| hsa-miR-337-3p | 33.59 | 33.68 | 35.68 | 34.50 | 35.71 | 33.90 |
| hsa-miR-494 |  | 38.33 |  | 38.69 | 39.29 | 37.46 |
| hsa-miR-371-3p |  |  |  |  |  |  |
| hsa-miR-103 | 26.48 | 25.91 | 27.52 | 26.23 | 26.57 | 25.12 |
| hsa-miR-144 | 27.33 | 26.73 | 27.11 | 26.08 | 26.26 | 24.73 |
| hsa-miR-184 |  |  |  |  |  |  |
| hsa-miR-631 |  | 38.14 |  | 36.23 |  | 39.08 |
| hsa-miR-519a | 39.47 |  |  |  | 39.19 | 39.48 |
| hsa-miR-211 | 35.02 | 37.72 | 36.06 | 37.82 | 37.21 | 36.65 |
| hsa-miR-802 | 37.57 | 39.14 |  |  |  |  |
| hsa-let-7f | 26.67 | 27.79 | 28.91 | 29.60 | 28.04 | 28.73 |
| hsa-miR-625* | 32.02 | 32.09 | 32.30 | 32.19 | 32.87 | 32.15 |
| hsa-miR-34a | 32.54 | 29.79 | 35.37 | 32.43 | 36.31 | 33.20 |
| hsa-miR-744 | 32.85 | 32.26 | 33.82 | 32.66 | 33.25 | 31.88 |
| hsa-miR-518e |  | 37.70 |  | 38.57 |  | 37.93 |
| hsa-miR-29b | 31.56 | 29.32 | 32.32 | 30.21 | 32.02 | 29.52 |
| hsa-miR-658 |  |  |  |  |  |  |
| hsa-miR-572 | 37.63 | 37.21 | 37.04 | 35.74 | 36.87 | 35.36 |
| hsa-let-7a | 28.92 | 29.06 | 30.70 | 30.76 | 30.91 | 30.27 |
| hsa-miR-30e | 29.15 | 29.39 | 30.67 | 30.56 | 30.08 | 29.72 |
| hsa-miR-433 | 35.47 | 35.37 | 37.92 | 36.00 | 36.01 | 34.11 |
| hsa-miR-660 | 31.94 | 32.58 | 33.82 |  | 32.81 | 31.65 |
| hsa-let-7c | 29.36 | 29.88 | 33.03 | 31.93 | 33.99 | 31.63 |
| hsa-miR-28-5p | 29.13 | 29.39 | 31.64 | 31.42 | 31.04 | 30.34 |
| hsa-miR-324-5p | 31.06 | 30.86 | 31.74 | 31.18 | 31.22 | 29.92 |
| hsa-miR-219-5p | 34.27 | 32.44 | 37.46 | 34.20 | 38.59 | 33.60 |
| hsa-miR-19b | 23.77 | 24.46 | 24.57 | 24.96 | 23.73 | 23.85 |
| hsa-miR-526b |  |  |  |  |  | 37.03 |
| hsa-miR-720 | 26.49 | 24.20 | 28.12 | 24.95 | 29.53 | 25.17 |
| hsa-miR-30b | 24.85 | 25.60 | 27.46 | 27.89 | 27.18 | 27.46 |
| hsa-miR-637 |  |  |  | 39.22 |  |  |
| hsa-miR-422a |  | 38.68 |  | 38.45 |  |  |
| hsa-miR-199a-3p | 28.40 | 27.47 | 29.03 | 27.88 | 28.99 | 26.79 |
| hsa-miR-335 | 28.13 | 28.96 | 29.06 | 29.49 | 29.40 | 29.15 |
| hsa-miR-134 | 32.01 | 32.25 | 32.91 | 33.53 | 31.62 | 31.33 |
| hsa-miR-21 | 23.77 | 23.89 | 24.92 | 24.95 | 24.96 | 24.55 |
| hsa-miR-129-3p |  | 37.41 |  |  |  |  |
| hsa-miR-26b | 26.94 | 27.13 | 29.07 | 29.06 | 28.48 | 28.00 |
| hsa-miR-214 | 35.43 | 35.96 | 35.34 | 36.26 | 36.00 | 35.10 |
| hsa-miR-32 | 30.61 | 27.31 | 31.16 | 28.38 | 30.51 | 27.46 |
| hsa-miR-324-3p | 30.83 | 30.04 | 31.62 | 30.66 | 31.00 | 29.38 |
| hsa-miR-488 |  |  |  |  |  |  |
| hsa-miR-371-5p |  |  |  | 38.73 |  | 38.02 |
| hsa-miR-455-5p | 31.31 | 31.55 | 34.58 | 34.51 | 37.02 | 36.65 |
| hsa-miR-891a |  |  |  | 38.19 |  |  |
| hsa-miR-549 |  |  |  |  |  |  |
| hsa-miR-205 | 34.54 | 33.92 | 35.57 | 33.02 | 33.82 | 32.89 |
| hsa-miR-518b |  | 38.30 |  | 38.24 |  |  |
| hsa-miR-361-5p | 35.91 | 36.65 | 37.45 | 36.98 | 36.24 | 34.45 |
| hsa-miR-454 | 34.63 | 35.16 | 35.32 | 35.83 | 35.12 | 33.54 |
| hsa-miR-15a | 26.23 | 25.39 | 26.61 | 25.31 | 25.65 | 24.07 |
| hsa-miR-191 | 27.19 | 28.77 | 28.08 | 29.47 | 27.19 | 28.27 |
| hsa-miR-608 |  | 37.63 |  |  |  |  |
| hsa-miR-576-5p | 34.42 | 33.53 | 36.13 | 35.39 | 35.98 | 34.91 |
| hsa-miR-497 | 33.81 | 29.88 | 35.75 | 30.88 | 34.56 | 30.25 |
| hsa-miR-19a | 28.02 | 29.30 | 28.78 | 29.26 | 28.59 | 28.10 |
| hsa-miR-187* |  | 37.24 |  |  |  |  |
| hsa-miR-620 |  |  |  |  |  |  |
| hsa-let-7i | 29.78 | 28.63 | 30.17 | 28.80 | 28.78 | 27.71 |
| hsa-miR-501-5p | 34.28 | 34.10 | 34.50 | 34.93 | 34.76 | 34.26 |
| hsa-miR-652 | 29.50 | 28.28 | 30.20 | 28.41 | 29.79 | 27.53 |
| hsa-miR-1979 | 27.79 | 27.38 | 29.33 | 28.64 | 29.39 | 27.56 |
| hsa-miR-30e* | 28.62 | 28.96 | 31.10 | 30.98 | 31.13 | 30.52 |
| hsa-miR-181c | 38.01 | 33.25 | 38.45 | 34.12 | 36.43 | 33.12 |
| hsa-miR-499-5p | 39.38 | 33.72 |  | 36.20 |  | 36.12 |
| hsa-miR-548c-3p |  |  |  |  |  |  |
| hsa-miR-152 | 28.94 | 29.41 | 30.08 | 30.38 | 30.34 | 29.84 |
| hsa-miR-93 | 25.72 | 26.06 | 26.25 | 26.26 | 25.66 | 25.06 |
| hsa-miR-490-3p | 35.61 | 32.91 | 36.24 | 37.25 | 35.75 | 36.37 |
| hsa-miR-29c | 29.49 | 27.59 | 31.26 | 28.62 | 30.31 | 27.61 |
| hsa-miR-372 |  |  |  |  |  |  |
| hsa-miR-133a | 35.29 | 33.19 | 35.92 | 33.15 | 35.90 | 32.79 |
| hsa-miR-124 | 36.67 | 34.06 |  | 33.74 | 38.43 | 33.66 |
| hsa-miR-190 | 36.81 | 36.98 | 38.29 | 35.33 |  | 34.07 |
| hsa-miR-302a |  |  |  | 38.05 | 39.91 | 39.55 |
| hsa-miR-595 |  | 38.22 | 39.54 | 37.27 | 37.04 | 38.80 |
| hsa-miR-602 | 38.99 | 34.23 | 39.08 | 34.18 |  | 32.80 |
| hsa-miR-223 | 22.06 | 22.57 | 22.61 | 22.80 | 21.74 | 21.48 |
| hsa-miR-627 | 33.70 | 34.71 | 34.49 | 35.42 | 33.83 | 34.06 |
| hsa-miR-34b |  | 37.06 | 39.50 | 38.29 |  |  |
| hsa-miR-410 | 32.70 | 32.38 | 33.55 | 32.68 | 32.59 | 30.71 |
| hsa-miR-17 | 28.33 | 29.21 | 29.47 | 29.77 | 28.93 | 28.56 |
| hsa-miR-376a | 29.68 | 30.02 | 31.39 | 30.37 | 29.22 | 29.09 |
| hsa-miR-877 | 34.24 | 33.98 | 35.02 | 34.48 | 34.26 | 35.14 |
| hsa-miR-512-5p |  |  |  |  |  |  |
| hsa-miR-449a |  | 37.34 | 36.07 |  | 37.29 |  |
| hsa-miR-498 |  |  |  | 38.33 |  |  |
| hsa-miR-148b | 27.88 | 27.31 | 28.63 | 27.61 | 28.27 | 27.13 |
| hsa-miR-127-3p | 36.45 | 33.39 | 34.55 | 33.52 | 32.17 | 30.71 |
| hsa-miR-598 | 31.17 | 32.29 | 32.39 | 32.10 | 32.31 | 31.88 |
| hsa-miR-96 | 39.07 | 35.70 | 37.26 | 36.03 | 37.36 | 35.65 |
| hsa-let-7d | 30.73 | 31.05 | 32.54 | 31.64 | 31.22 | 30.59 |
| hsa-miR-135b |  |  |  |  |  |  |
| hsa-miR-495 | 32.03 | 30.92 | 32.59 | 31.59 | 31.81 | 29.28 |
| hsa-miR-299-5p | 36.89 | 39.28 |  | 39.85 | 35.55 | 39.11 |
| hsa-miR-34c-3p |  | 37.23 | 38.69 | 37.15 | 39.16 | 37.00 |
| hsa-miR-596 |  | 38.16 |  | 36.78 |  | 38.78 |
| hsa-miR-126* | 26.23 | 27.52 | 27.54 | 27.23 | 29.59 | 26.24 |
| hsa-miR-145 | 29.44 | 29.64 | 29.72 | 30.19 | 29.26 | 29.01 |
| SNORD38B |  | 36.50 |  |  | 35.22 | 33.08 |
| hsa-miR-516a-5p |  |  |  |  |  |  |
| hsa-miR-421 | 31.22 | 32.17 | 32.15 | 32.48 | 31.42 | 32.00 |
| hsa-miR-96* |  |  |  |  |  |  |
| hsa-miR-362-5p | 35.13 | 37.20 | 37.48 | 38.00 | 38.40 | 37.55 |
| hsa-miR-615-3p | 36.18 | 37.03 | 37.32 | 36.86 | 37.93 | 36.10 |
| hsa-miR-550a | 34.92 | 36.23 |  | 37.00 | 37.09 | 36.59 |
| hsa-miR-766 | 30.76 | 30.73 | 31.16 | 31.20 | 30.49 | 30.16 |
| hsa-miR-200b | 33.84 | 33.05 | 34.80 | 35.26 | 36.41 | 34.89 |
| hsa-miR-298 |  | 36.41 |  |  |  |  |
| hsa-miR-193a-5p | 33.04 | 33.52 | 36.27 | 35.28 | 36.53 | 35.32 |
| hsa-miR-449b |  | 38.01 |  |  |  |  |
| hsa-miR-520d-5p |  |  |  |  | 36.69 |  |
| hsa-miR-192 | 23.62 | 23.68 | 27.16 | 27.07 | 30.04 | 29.37 |
| hsa-miR-29a | 29.16 | 27.77 | 30.93 | 29.29 | 31.22 | 28.57 |
| hsa-miR-18a* | 33.79 | 33.42 | 34.42 | 34.37 | 33.85 | 33.25 |
| hsa-miR-383 |  |  |  |  |  |  |
| hsa-miR-9 | 38.11 | 38.65 | 37.30 |  | 37.72 | 37.71 |
| hsa-miR-202* | 38.32 | 38.37 | 38.49 | 39.81 | 37.99 | 38.77 |
| hsa-miR-363* | 38.18 |  | 36.38 | 39.61 |  |  |
| hsa-miR-147b |  | 38.45 | 39.73 | 37.94 |  | 38.02 |
| hsa-miR-197 | 28.38 | 29.10 | 29.49 | 29.92 | 29.21 | 28.87 |
| hsa-miR-597 |  | 39.25 |  | 39.11 |  | 38.32 |
| hsa-miR-326 | 34.24 | 28.51 | 34.27 | 28.51 | 33.79 | 28.22 |
| hsa-miR-15b | 26.04 | 26.86 | 26.70 | 27.16 | 26.13 | 26.04 |
| hsa-miR-105 |  |  |  |  |  |  |
| hsa-miR-196b | 35.34 | 37.08 | 34.34 | 36.06 | 34.84 | 33.68 |
| hsa-miR-296-5p | 37.37 | 33.38 |  | 33.76 | 36.05 | 32.49 |
| hsa-miR-20b | 32.44 | 33.50 | 32.55 | 32.95 | 32.28 | 31.69 |
| hsa-miR-147 |  |  |  |  |  |  |
| hsa-miR-198 |  |  |  |  |  |  |
| hsa-miR-375 | 30.01 | 31.33 | 32.30 | 33.08 | 32.56 | 34.30 |
| hsa-miR-517a |  |  |  |  |  |  |
| hsa-miR-361-3p | 34.05 | 31.69 | 35.83 | 33.63 | 35.56 | 32.38 |
| hsa-miR-21* | 39.18 | 33.21 | 39.77 | 33.97 | 38.05 | 33.30 |
| hsa-miR-220a |  |  |  |  |  |  |
| hsa-miR-518f |  |  |  | 36.70 | 36.87 | 36.64 |
| hsa-miR-222 | 28.56 | 28.47 | 29.14 | 28.77 | 28.76 | 28.01 |
| hsa-miR-617 |  |  |  |  |  |  |
| hsa-miR-154 | 31.19 | 31.63 | 32.65 | 32.31 | 31.60 | 30.37 |
| hsa-miR-708 | 36.28 |  |  |  |  | 38.21 |
| hsa-let-7b | 26.36 | 26.91 | 27.47 | 27.91 | 26.63 | 26.39 |
| hsa-miR-95 | 32.60 | 32.75 | 34.75 | 34.86 | 36.37 | 33.99 |
| hsa-miR-517c |  |  |  | 38.29 | 38.70 |  |
| hsa-miR-151-5p | 27.35 | 27.72 | 29.21 | 28.32 | 28.62 | 27.26 |
| hsa-miR-502-5p | 37.51 | 35.02 | 37.01 | 35.61 | 37.21 | 34.14 |
| hsa-miR-345 | 32.08 | 30.36 | 34.85 | 31.83 | 35.33 | 31.60 |
| hsa-miR-509-3p | 37.38 | 35.27 | 38.28 | 36.38 |  | 36.34 |
| U6 | 34.94 | 33.11 | 34.26 | 33.75 | 35.70 | 33.47 |
| hsa-miR-382 | 31.02 | 32.17 | 31.43 | 32.27 | 30.23 | 30.54 |
| hsa-miR-373 |  | 39.94 |  | 39.44 | 38.17 | 39.94 |
| hsa-miR-200c | 33.39 | 34.26 | 34.67 | 34.97 | 33.04 | 33.44 |
| hsa-miR-9* | 35.23 |  | 36.38 | 37.29 | 37.13 | 37.12 |
| hsa-miR-181b | 33.60 | 33.53 | 34.94 | 34.77 | 34.77 | 33.68 |
| hsa-miR-628-3p | 32.39 | 32.96 | 33.00 | 32.75 | 32.61 | 32.22 |
| hsa-miR-195 | 29.91 | 30.53 | 31.99 | 31.86 | 31.03 | 31.38 |
| hsa-miR-183 | 38.23 | 37.02 | 37.41 | 36.60 | 36.83 | 36.56 |
| hsa-miR-135a | 36.68 | 37.93 | 37.46 | 37.64 | 36.45 | 37.27 |
| hsa-miR-30b* | 36.79 | 35.64 |  | 37.44 | 38.65 | 35.89 |
| hsa-miR-146b-5p | 31.29 | 30.61 | 33.22 | 32.37 | 33.44 | 31.96 |
| hsa-miR-301a | 30.75 | 30.87 | 31.28 | 31.50 | 30.62 | 29.93 |
| hsa-miR-1 | 32.27 | 32.49 | 32.33 | 33.40 | 32.12 | 31.53 |
| hsa-miR-299-3p |  | 38.04 |  | 37.03 |  | 35.57 |
| hsa-miR-142-3p | 27.07 | 27.01 | 27.45 | 26.97 | 26.23 | 25.60 |
| hsa-miR-338-3p | 31.10 | 29.58 | 32.07 | 30.28 | 31.48 | 29.13 |
| hsa-miR-584 | 30.18 | 31.92 | 31.00 | 31.88 | 32.27 | 31.37 |
| hsa-miR-377 | 36.32 | 31.75 |  | 31.63 | 38.42 | 30.09 |
| hsa-miR-216a | 37.27 | 34.99 |  | 36.63 |  | 36.08 |
| hsa-miR-206 |  |  |  |  |  |  |
| hsa-miR-921 |  |  |  |  |  |  |
| hsa-miR-513a-5p |  |  |  |  |  | 38.00 |
| hsa-miR-140-3p | 27.92 | 28.05 | 29.07 | 28.32 | 27.88 | 27.65 |
| hsa-miR-181a | 29.59 | 29.51 | 30.64 | 30.25 | 30.19 | 28.87 |
| hsa-miR-122 | 20.50 | 21.37 | 24.12 | 24.34 | 28.75 | 28.40 |
| hsa-miR-106a | 24.52 | 25.54 | 25.43 | 25.86 | 25.13 | 24.62 |
| hsa-miR-182 | 34.80 | 36.65 | 36.28 | 35.81 | 34.95 | 34.03 |
| hsa-miR-370 | 36.08 | 34.48 | 38.11 | 35.10 | 34.45 | 31.62 |
| hsa-let-7d* | 27.44 | 28.33 | 28.58 | 28.82 | 28.46 | 27.84 |
| hsa-miR-425* | 30.64 | 30.42 | 31.61 | 30.60 | 31.12 | 29.49 |
| hsa-miR-450a | 36.68 | 37.98 | 37.97 | 38.96 | 35.23 | 36.78 |
| hsa-miR-411 | 35.64 | 35.59 | 37.14 | 36.01 | 32.94 | 33.30 |
| hsa-miR-216b | 38.25 | 38.79 |  | 38.82 |  | 38.33 |
| hsa-miR-106b | 30.98 | 30.10 | 31.14 | 30.48 | 31.05 | 29.36 |
| hsa-miR-886-3p |  | 33.54 |  | 33.93 | 37.38 | 33.95 |
| hsa-miR-510 |  | 34.32 |  | 33.73 |  | 33.78 |
| hsa-miR-212 | 35.24 | 36.87 | 35.65 | 36.89 | 36.87 | 36.31 |
| hsa-miR-525-5p |  | 38.53 |  | 37.13 |  | 38.11 |
| hsa-miR-589 | 31.02 | 34.93 | 33.93 | 36.23 | 36.29 | 34.73 |
| hsa-miR-576-3p | 36.56 |  | 39.06 | 39.26 | 36.77 | 36.35 |
| hsa-miR-583 |  |  |  | 37.95 |  |  |
| hsa-miR-483-3p | 30.86 | 30.56 | 35.25 | 32.79 | 36.96 | 34.24 |
| hsa-miR-582-5p | 33.20 | 34.26 | 36.98 | 36.95 | 35.79 | 34.53 |
| hsa-miR-886-5p | 35.70 | 36.11 | 36.69 | 35.66 | 37.97 | 34.85 |
| hsa-miR-33b | 35.06 | 28.73 | 38.21 | 30.49 |  | 31.11 |
| hsa-miR-193a-3p | 39.57 | 33.39 |  | 35.25 |  |  |
| hsa-miR-153 | 39.06 | 35.14 | 38.35 | 36.17 | 37.23 | 34.94 |
| Blank (H20) |  |  |  |  |  |  |
| hsa-miR-409-3p | 30.57 | 31.11 | 31.84 | 31.36 | 30.27 | 29.29 |
| hsa-miR-22 | 29.58 | 26.99 | 31.30 | 27.26 | 31.66 | 27.01 |
| hsa-miR-629 | 33.22 | 33.94 | 34.18 | 33.43 | 33.31 | 31.75 |
| hsa-miR-365 | 28.48 | 27.63 | 31.70 | 30.47 | 33.63 | 31.22 |
| hsa-miR-429 | 35.44 | 38.03 | 36.26 | 38.89 | 37.42 | 37.01 |
| hsa-miR-98 | 33.87 | 34.73 | 35.18 | 35.56 | 34.99 | 33.70 |
| hsa-miR-518a-3p |  |  |  | 37.95 |  |  |
| hsa-miR-137 | 39.47 | 39.05 | 39.33 | 38.48 |  | 38.98 |
| hsa-miR-508-3p |  | 39.70 |  |  |  |  |
| hsa-miR-539 | 39.10 |  | 38.72 | 38.38 | 37.12 | 36.33 |
| hsa-miR-148a | 26.36 | 25.92 | 29.13 | 28.04 | 30.48 | 28.05 |
| hsa-miR-146a | 28.36 | 28.10 | 28.68 | 28.07 | 28.80 | 27.37 |
| hsa-miR-139-5p | 30.31 | 32.25 | 31.01 | 31.87 | 30.47 | 30.33 |
| hsa-miR-373* | 36.32 | 33.48 | 36.88 | 33.17 | 38.87 | 32.67 |
| hsa-miR-149 | 34.72 |  |  |  |  | 37.36 |
| hsa-miR-642a | 37.64 | 38.10 |  | 37.29 |  | 36.31 |
| hsa-miR-31 | 37.31 | 32.50 |  | 32.04 | 37.01 | 31.98 |
| hsa-miR-451 | 21.77 | 21.28 | 22.03 | 20.40 | 20.57 | 18.72 |
| hsa-miR-100 | 28.69 | 30.22 | 32.96 | 33.34 |  | 37.72 |
| hsa-miR-27b | 25.04 | 25.53 | 26.31 | 26.45 | 27.02 | 25.84 |
| hsa-miR-523 |  |  |  |  |  |  |
| Blank (H20) |  |  |  |  |  |  |

B,

|  | Human panel **II** V2.M/R, raw C_T_ values | | | | | |
| --- | --- | --- | --- | --- | --- | --- |
|  | **HBeAg positive pool** | | **HBeAg negative pool** | | **Control pool** | |
| **miRNA** | Replicate 1 | Replicate 2 | Replicate 1 | Replicate 2 | Replicate 1 | Replicate 2 |
| hsa-miR-499-3p | 36.93 | 36.36 |  |  |  |  |
| hsa-miR-219-1-3p |  | 38.00 |  | 39.05 |  |  |
| hsa-miR-543 | 31.94 | 31.64 | 32.39 | 32.61 | 31.56 | 30.67 |
| hsa-miR-1245 |  |  |  |  |  |  |
| hsa-miR-522 |  |  |  |  |  |  |
| hsa-miR-571 | 35.69 | 35.30 | 35.74 | 36.41 | 37.04 | 34.14 |
| hsa-miR-323-5p | 39.75 | 39.44 | 36.83 | 37.39 | 37.29 | 38.40 |
| hsa-miR-592 | 37.86 | 38.50 |  | 39.71 |  |  |
| hsa-miR-487a | 35.37 | 35.25 | 35.81 | 36.06 | 35.47 | 34.32 |
| hsa-miR-1249 | 33.16 | 34.22 | 34.52 | 34.49 | 34.56 | 33.21 |
| hsa-miR-25* |  | 39.28 | 37.18 | 37.31 |  |  |
| hsa-miR-922 |  |  |  |  |  |  |
| hsa-miR-124* |  |  |  |  |  |  |
| hsa-miR-1264 |  |  |  |  |  |  |
| hsa-miR-504 | 38.84 | 38.22 | 39.13 |  | 39.95 | 38.59 |
| hsa-miR-138-1* |  | 39.55 | 39.09 | 38.56 |  |  |
| hsa-miR-502-3p | 32.21 | 32.57 | 32.75 | 33.40 | 32.71 | 32.37 |
| hsa-miR-490-5p |  |  | 38.70 | 36.97 |  |  |
| hsa-miR-567 | 37.29 | 36.72 |  | 36.66 | 37.05 |  |
| hsa-miR-18b* | 38.79 |  | 37.81 | 38.50 |  |  |
| hsa-miR-125a-3p | 37.24 | 39.59 | 37.42 |  |  | 36.02 |
| hsa-miRPlus-A1027 | 34.88 | 35.20 | 35.08 | 35.54 | 35.76 | 34.88 |
| hsa-miR-129* |  |  |  |  |  |  |
| hsa-miR-148a* | 33.09 | 33.26 | 37.55 | 35.21 | 35.99 | 35.59 |
| hsa-miR-412 |  |  |  |  |  |  |
| Blank (H20) |  |  |  |  |  |  |
| hsa-miRPlus-C1066 | 35.25 | 35.82 | 34.42 | 35.36 | 35.83 | 34.66 |
| hsa-miR-548o | 37.14 | 36.28 | 36.73 | 36.42 | 36.79 | 37.67 |
| hsa-miR-143* | 35.03 | 35.11 | 35.31 | 34.33 | 35.26 | 34.21 |
| hsa-miR-513a-3p |  |  | 39.15 |  |  | 37.78 |
| hsa-miR-943 | 36.31 | 35.85 | 36.44 | 37.47 | 38.92 | 35.75 |
| hsa-miR-196b* | 37.23 | 38.67 | 35.36 | 36.26 |  | 35.37 |
| hsa-miR-200b* | 34.03 | 34.18 | 34.77 | 34.60 | 34.72 | 34.02 |
| hsa-miR-551b* | 39.00 |  |  | 38.89 |  | 38.32 |
| hsa-miR-942 | 35.33 | 38.48 | 37.01 | 36.01 | 34.54 | 33.61 |
| hsa-miR-515-3p | 39.03 | 38.64 |  |  |  | 38.91 |
| hsa-miRPlus-C1076 |  | 37.92 |  |  |  | 39.61 |
| hsa-miR-653 | 37.43 | 36.49 | 37.10 | 36.06 | 36.96 | 37.81 |
| hsa-miR-24-2* | 33.68 | 33.62 | 33.89 | 34.30 | 33.28 | 33.98 |
| hsa-miR-1539 | 36.98 | 37.13 | 35.67 | 37.37 | 35.75 | 36.48 |
| hsa-miR-27b* | 36.31 | 36.22 | 37.93 | 39.23 | 39.51 | 37.23 |
| hsa-miR-1909 | 28.00 | 27.37 | 27.84 | 28.30 | 27.87 | 27.43 |
| hsa-miR-770-5p | 36.27 | 36.48 |  | 37.17 | 36.26 | 37.18 |
| hsa-miR-548l |  | 38.34 | 38.21 | 38.93 |  | 39.59 |
| hsa-miR-376a* |  |  | 38.17 | 37.48 | 36.95 | 36.04 |
| hsa-miR-1247 | 33.66 | 33.84 | 37.10 | 37.10 | 37.68 | 39.14 |
| hsa-miR-520b |  |  | 39.05 |  |  | 38.72 |
| hsa-miR-936 | 39.30 | 37.98 |  | 38.60 |  | 38.75 |
| hsa-miR-28-3p | 28.83 | 28.66 | 30.47 | 30.40 | 30.52 | 29.88 |
| hsa-miR-875-5p | 38.88 |  |  |  |  |  |
| hsa-miR-551a | 34.74 | 36.77 | 35.46 | 34.55 | 33.94 | 33.32 |
| hsa-miR-1183 |  | 39.12 | 37.73 | 39.15 |  | 37.15 |
| hsa-miR-758 | 36.83 | 36.40 | 37.34 | 37.35 | 36.78 | 35.53 |
| hsa-miR-1244 | 38.50 | 39.21 | 39.56 |  |  | 39.34 |
| hsa-miR-566 | 39.57 |  |  | 39.98 |  |  |
| hsa-miR-1256 |  | 37.71 | 36.86 | 38.13 |  |  |
| hsa-miR-516b* |  |  |  |  |  |  |
| hsa-miR-548c-5p | 33.88 | 33.76 | 32.99 | 33.21 | 32.31 | 32.27 |
| hsa-miR-496 | 33.03 | 32.80 | 33.71 | 34.03 | 33.30 | 32.07 |
| hsa-miR-876-3p |  | 38.29 |  |  |  |  |
| hsa-miR-532-3p | 31.60 | 31.65 | 32.25 | 32.57 | 31.74 | 31.83 |
| hsa-miR-654-3p | 32.61 | 32.58 | 32.53 | 32.73 | 32.25 | 31.88 |
| hsa-miR-659 |  | 38.63 | 39.96 | 38.72 |  | 39.72 |
| hsa-miR-135b* |  | 37.65 |  | 39.29 |  |  |
| hsa-miR-641 | 37.14 | 34.88 |  | 37.55 |  | 37.55 |
| hsa-miR-616* | 34.13 | 35.80 | 37.05 | 36.07 | 36.06 | 35.14 |
| hsa-miR-489 |  |  |  |  |  |  |
| hsa-miR-335* |  |  |  |  | 39.09 | 39.28 |
| hsa-miR-892a | 37.17 | 34.77 | 35.43 | 37.43 | 35.70 | 34.86 |
| hsa-miR-10b* |  |  |  |  |  |  |
| hsa-miR-122* | 27.53 | 27.44 | 30.85 | 30.73 | 37.90 |  |
| hsa-miR-100* | 39.37 |  |  |  |  |  |
| hsa-miR-769-3p | 39.80 | 36.35 | 37.34 |  | 36.82 | 37.21 |
| UniSp6 CP | 26.51 | 27.22 | 26.22 | 28.04 | 26.52 | 27.41 |
| hsa-miR-519c-5p | 36.71 | 36.15 | 35.21 | 38.64 | 37.12 | 35.76 |
| hsa-miR-938 |  | 39.29 | 39.43 |  |  |  |
| hsa-miR-937 | 36.49 | 35.91 | 36.66 | 35.67 | 35.98 | 38.42 |
| hsa-miR-1911* | 39.27 | 38.75 |  |  |  | 39.35 |
| hsa-miR-640 | 34.99 | 37.62 | 36.00 | 34.55 | 34.91 | 34.90 |
| (hsa-miR-220b) | 38.43 |  | 37.79 |  |  | 36.30 |
| hsa-miR-29c* | 32.15 | 32.56 | 34.16 | 34.57 | 33.79 | 33.34 |
| hsa-miR-449b* | 35.41 | 35.67 | 37.61 | 36.98 | 37.59 | 38.96 |
| hsa-let-7f-1* | 32.73 | 32.96 | 34.29 | 33.29 | 34.56 | 32.98 |
| hsa-miR-297 | 35.28 | 34.63 | 35.13 | 37.08 | 35.62 | 35.46 |
| hsa-miRPlus-A1031 | 37.86 | 38.55 | 38.12 | 39.38 | 36.28 | 33.41 |
| hsa-miR-144* | 33.33 | 33.10 | 32.70 | 32.74 | 32.07 | 32.02 |
| hsa-miR-106a* | 36.17 | 39.40 | 37.96 | 37.63 | 38.25 | 37.85 |
| hsa-miR-1254 | 34.25 | 34.18 | 34.98 | 34.38 | 35.52 | 33.93 |
| hsa-miR-580 | 38.13 |  | 39.46 | 38.35 | 38.59 | 36.95 |
| hsa-miR-1252 |  | 39.66 |  |  |  |  |
| hsa-miR-643 | 36.33 | 36.48 | 38.02 | 36.86 | 38.09 | 36.19 |
| hsa-miR-30c-1* | 34.66 | 34.48 | 35.85 | 36.19 | 34.40 | 35.59 |
| hsa-miR-221* | 35.32 | 35.84 | 35.38 | 35.46 | 35.25 | 33.94 |
| hsa-miR-103-as | 37.34 | 35.27 | 38.27 | 38.03 | 37.03 | 35.63 |
| hsa-miR-411* | 36.62 | 35.70 | 36.92 | 36.87 | 36.25 | 35.44 |
| hsa-miR-519e* |  |  | 39.15 |  |  | 39.49 |
| hsa-miR-338-5p | 36.33 | 36.37 | 37.77 | 39.09 | 37.75 | 35.82 |
| hsa-miRPlus-C1089 | 39.11 | 38.40 | 37.24 | 39.69 |  |  |
| hsa-miR-323b-5p |  | 38.73 |  |  |  | 37.60 |
| hsa-miR-548i | 36.19 | 36.19 | 38.00 | 36.82 | 36.27 | 39.88 |
| hsa-miR-541 |  |  |  |  |  |  |
| hsa-miR-1272 | 34.55 | 34.23 | 34.55 | 36.02 | 35.56 | 34.30 |
| hsa-miR-1205 | 36.22 | 35.61 | 35.67 | 36.49 | 35.54 | 36.51 |
| hsa-miR-1266 | 35.25 | 35.95 | 36.16 | 37.13 | 35.79 | 34.54 |
| hsa-miR-431* | 35.38 | 35.47 | 36.58 | 37.66 | 34.88 | 35.50 |
| hsa-miR-621 | 36.06 | 36.41 | 37.17 | 37.50 | 36.16 | 35.77 |
| hsa-miR-556-5p | 39.23 |  |  |  | 37.46 | 38.13 |
| hsa-miR-1267 | 37.03 | 36.11 | 37.42 | 37.52 | 38.37 | 34.05 |
| hsa-miR-141* | 37.00 | 38.74 | 38.21 | 39.28 | 38.03 | 36.42 |
| hsa-miR-1269 | 39.32 | 37.45 | 37.54 |  |  | 36.70 |
| hsa-miR-501-3p | 33.42 | 32.83 | 33.59 | 33.67 | 32.88 | 33.04 |
| hsa-miR-15b* | 31.32 | 31.32 | 31.77 | 31.35 | 31.58 | 30.88 |
| hsa-miR-146b-3p | 34.10 | 34.58 | 37.76 | 35.79 |  | 35.56 |
| hsa-miR-222* | 37.54 | 38.61 | 38.55 |  |  | 38.57 |
| hsa-miR-601 | 38.41 |  |  |  |  |  |
| hsa-miR-924 |  |  | 39.72 |  |  |  |
| hsa-miR-29a* | 31.18 | 31.22 | 33.34 | 33.52 | 34.10 | 33.86 |
| hsa-let-7a-2* | 37.46 | 36.14 |  |  |  |  |
| hsa-miR-520f |  |  |  |  |  |  |
| hsa-miR-101* | 34.77 | 34.30 | 36.71 | 36.37 | 36.04 | 36.10 |
| hsa-miR-520a-3p |  |  |  |  |  |  |
| hsa-miR-423-5p | 28.20 | 28.21 | 28.45 | 28.69 | 28.43 | 27.85 |
| hsa-miR-517* | 37.12 | 38.91 | 37.28 | 38.82 |  | 39.06 |
| hsa-miR-380 | 36.70 | 38.28 | 38.93 | 37.41 | 37.80 | 36.47 |
| hsa-miR-1296 | 34.27 | 34.29 | 34.97 | 35.58 | 34.95 | 34.11 |
| hsa-miR-2053 |  |  |  |  |  |  |
| hsa-miR-920 | 35.77 | 36.99 | 36.19 |  | 37.87 |  |
| hsa-miR-1471 | 38.59 | 37.18 | 38.09 | 38.37 | 38.06 | 37.22 |
| hsa-miR-19b-2* | 36.76 |  |  |  |  |  |
| hsa-miR-10a* | 39.25 | 39.10 | 39.74 | 39.33 | 39.99 | 36.48 |
| hsa-miR-106b* | 32.11 | 31.92 | 32.46 | 32.75 | 31.23 | 31.33 |
| hsa-miR-365* |  | 39.36 | 37.87 |  | 37.93 |  |
| hsa-miR-619 |  | 36.48 |  | 39.87 | 39.49 | 35.60 |
| hsa-miR-518f* |  | 37.43 | 39.18 | 39.45 |  | 35.48 |
| hsa-miR-17* | 29.32 | 29.58 | 33.84 | 30.93 | 30.43 | 30.19 |
| hsa-miR-2113 | 36.14 | 34.57 | 36.10 | 37.02 | 36.85 | 36.70 |
| hsa-miR-877* | 34.15 | 34.72 | 34.17 | 35.40 | 33.82 | 34.40 |
| hsa-miR-362-3p | 36.46 | 35.72 | 37.19 | 36.67 | 37.30 | 36.94 |
| hsa-miR-3180-3p | 35.89 | 36.27 | 35.14 | 35.50 | 37.06 | 36.04 |
| hsa-miR-7-1* | 35.50 | 34.40 | 36.26 | 36.13 | 36.36 | 35.99 |
| hsa-miR-767-5p |  |  |  |  |  |  |
| hsa-miR-562 |  | 37.09 | 37.30 |  | 38.54 |  |
| hsa-miRPlus-D1033 | 33.34 | 33.24 | 35.93 | 36.04 | 35.85 | 35.70 |
| hsa-miR-300 | 36.10 | 36.02 | 36.31 | 37.01 | 36.50 | 36.27 |
| hsa-miR-604 | 39.54 | 37.83 | 36.47 | 38.66 | 38.37 | 36.49 |
| hsa-miR-130b* | 32.96 | 33.38 | 33.99 | 34.32 | 33.51 | 33.01 |
| hsa-miR-149* | 35.09 | 34.19 | 34.72 | 34.30 | 34.91 | 34.74 |
| hsa-miR-1271 | 35.68 | 35.77 | 37.04 | 36.52 | 37.24 | 35.17 |
| hsa-miR-520h | 36.67 | 36.75 | 35.63 | 36.73 | 36.90 | 35.79 |
| hsa-miR-769-5p | 33.50 | 33.68 | 34.19 | 34.33 | 33.05 | 33.70 |
| hsa-miR-612 |  | 39.44 |  |  |  |  |
| hsa-miR-1237 | 35.18 | 35.69 | 35.76 | 35.67 | 35.60 | 35.74 |
| hsa-miR-527 |  |  |  |  |  |  |
| hsa-miR-1260 | 32.16 | 31.91 | 33.03 | 33.29 | 33.63 | 33.04 |
| hsa-miR-182* | 36.28 | 37.00 | 37.70 | 37.05 | 35.53 | 35.36 |
| hsa-miR-632 | 29.37 | 29.17 | 28.93 | 30.32 | 29.78 | 29.26 |
| hsa-miR-508-5p |  |  |  |  |  | 36.33 |
| hsa-miR-671-3p | 34.89 | 35.51 | 35.61 | 37.09 | 34.97 | 34.66 |
| hsa-miR-941 | 34.17 | 34.26 | 35.72 | 35.32 | 35.11 | 34.79 |
| hsa-miR-23b* | 35.28 | 37.13 | 36.26 |  | 36.60 | 36.20 |
| hsa-miR-591 | 39.00 |  | 39.07 |  |  |  |
| hsa-miR-26b* | 33.57 | 34.10 | 34.28 | 34.47 | 35.27 | 33.34 |
| hsa-miR-519b-3p |  |  |  |  | 39.83 |  |
| hsa-miR-30d* | 32.29 | 32.63 | 35.17 | 34.44 | 33.99 | 35.22 |
| hsa-miR-526a | 36.61 | 37.13 | 36.13 | 37.49 | 36.15 | 38.86 |
| hsa-miR-675b | 35.06 | 34.76 | 35.47 | 35.77 | 37.04 | 35.54 |
| hsa-miR-520e | 39.51 |  |  | 39.00 |  |  |
| hsa-miR-646 | 36.24 | 36.72 | 36.81 |  | 37.65 | 38.34 |
| hsa-miR-519e |  | 39.25 |  |  |  |  |
| hsa-miR-103 | 25.56 | 25.58 | 26.12 | 26.27 | 25.38 | 25.05 |
| hsa-miR-26a-1* | 35.04 | 34.18 | 35.50 | 35.01 | 34.65 | 34.43 |
| hsa-miR-1908 | 32.41 | 31.94 | 32.85 | 33.10 | 33.03 | 32.11 |
| hsa-miRPlus-D1061 |  | 39.69 |  | 39.26 |  |  |
| hsa-miR-103-2* | 31.82 | 31.80 | 32.55 | 32.24 | 31.76 | 31.18 |
| hsa-miR-586 |  |  |  |  |  |  |
| hsa-miR-1179 | 36.25 | 35.81 | 36.96 | 35.82 | 35.45 | 34.58 |
| hsa-miR-488* | 38.08 | 38.75 | 38.14 | 39.62 | 39.65 | 37.34 |
| hsa-miR-208b |  | 38.12 | 39.19 | 37.46 | 38.15 |  |
| hsa-miR-192* | 30.65 | 30.94 | 33.76 | 33.98 | 39.36 |  |
| hsa-miR-588 |  | 38.75 | 38.15 |  | 39.16 | 39.08 |
| hsa-miR-181a-2* | 33.32 | 33.92 | 35.64 | 34.53 | 36.32 | 36.54 |
| hsa-miR-1914 | 34.02 | 33.77 | 34.02 | 34.45 | 34.13 | 34.23 |
| hsa-miR-573 |  |  |  |  |  |  |
| hsa-miR-548d-5p | 38.13 | 39.14 | 38.85 | 38.50 | 37.67 | 36.73 |
| hsa-miR-194* | 32.98 | 32.74 | 34.21 | 34.05 | 34.89 | 34.32 |
| hsa-miR-1972 | 31.75 | 31.87 | 32.64 | 32.92 | 32.55 | 32.75 |
| hsa-miR-556-3p | 38.63 | 38.54 | 38.87 | 39.48 |  |  |
| hsa-miR-664 | 32.20 | 32.29 | 33.93 | 34.02 | 33.76 | 33.69 |
| hsa-let-7b* | 31.60 | 31.98 | 33.23 | 32.68 | 32.56 | 32.75 |
| hsa-miR-195* | 36.90 |  |  |  | 39.28 |  |
| hsa-miR-125b-1* | 35.29 | 35.60 | 39.54 | 38.25 | 39.48 | 38.94 |
| Blank (H20) |  |  |  |  |  |  |
| hsa-miR-519c-3p |  |  |  |  |  | 39.70 |
| hsa-miR-554 | 38.70 | 37.08 | 38.32 | 38.11 | 39.33 | 37.05 |
| hsa-miR-558 | 36.74 | 37.31 | 38.80 | 39.93 |  | 35.83 |
| hsa-miR-1243 |  |  |  |  |  |  |
| (hsa-miR-1201) |  | 37.44 | 37.99 | 38.55 | 39.18 | 36.50 |
| hsa-miR-1185 | 37.55 | 37.22 | 38.16 | 38.08 | 36.76 | 36.01 |
| hsa-miR-512-3p | 38.75 | 39.16 |  | 39.11 |  | 37.35 |
| hsa-miR-587 |  |  |  |  |  |  |
| hsa-miR-603 |  | 37.58 |  |  |  |  |
| hsa-miR-1184 |  |  |  |  |  |  |
| hsa-miR-20a* | 30.90 | 31.20 | 33.13 | 32.89 | 33.07 | 33.91 |
| hsa-miR-1263 |  |  |  |  |  |  |
| hsa-miR-455-3p | 27.07 | 27.14 | 30.51 | 30.80 | 33.10 | 32.86 |
| hsa-miR-582-3p | 38.22 | 37.31 | 38.09 | 38.21 | 37.11 | 37.44 |
| hsa-miR-409-5p | 36.59 | 37.55 | 36.42 | 36.47 | 35.71 | 35.58 |
| hsa-miR-452* |  |  | 38.96 |  |  |  |
| hsa-miR-19b-1* | 34.29 | 33.84 | 34.62 | 34.79 | 35.28 | 34.40 |
| hsa-miR-610 | 34.62 | 34.39 | 34.81 | 35.46 | 35.54 | 35.21 |
| hsa-miR-511 | 35.47 | 35.28 | 36.75 | 36.12 | 35.37 | 36.27 |
| hsa-miR-200c* |  |  |  |  | 37.06 |  |
| hsa-let-7a* | 32.96 | 32.64 | 33.72 | 34.16 | 33.99 | 33.07 |
| hsa-miR-135a* |  | 37.60 |  |  |  |  |
| hsa-miR-520a-5p |  |  | 39.34 |  |  |  |
| hsa-miR-1468 | 34.93 | 34.78 | 36.36 | 35.98 |  | 36.74 |
| hsa-miRPlus-C1100 |  |  |  |  |  |  |
| hsa-miR-191 | 28.43 | 28.95 | 29.00 | 28.91 | 28.41 | 27.95 |
| hsa-miR-145* | 33.48 | 34.39 | 34.44 | 35.17 | 34.87 | 34.73 |
| hsa-miR-548h | 37.11 | 35.53 | 35.66 | 36.87 | 37.55 | 37.08 |
| hsa-miR-378* | 31.30 | 31.10 | 33.82 | 33.65 | 36.05 | 34.79 |
| hsa-miR-553 |  |  |  |  |  |  |
| hsa-miR-181c* | 34.29 | 35.71 | 34.27 | 33.84 | 33.70 | 33.53 |
| hsa-miR-624* | 34.69 | 33.34 | 36.48 | 36.29 | 34.59 | 33.94 |
| hsa-miR-578 |  |  |  |  |  |  |
| hsa-miR-448 |  |  |  | 39.34 |  | 39.61 |
| hsa-miR-875-3p |  |  |  |  |  | 38.67 |
| hsa-miR-661 | 36.41 | 35.55 | 36.10 | 37.52 | 35.43 | 36.89 |
| hsa-miR-876-5p |  |  | 38.60 |  |  | 39.92 |
| hsa-miR-1913 | 31.46 | 31.77 | 32.10 | 32.40 | 31.89 | 31.78 |
| (hsa-miR-220c) |  |  |  |  |  |  |
| hsa-miR-218-1* | 39.59 | 38.45 | 39.46 |  | 38.64 |  |
| hsa-miR-744* | 34.28 | 34.72 | 35.11 | 33.78 | 34.32 | 33.53 |
| hsa-miR-548m |  |  |  |  |  |  |
| hsa-miR-138-2* | 37.94 | 37.69 | 37.54 | 37.71 |  | 38.38 |
| hsa-miR-561 |  |  |  |  |  |  |
| hsa-miR-99b* | 34.64 | 34.67 | 36.33 | 35.12 | 36.05 | 33.44 |
| hsa-miR-23a* | 38.33 | 37.59 |  | 37.70 | 39.43 | 37.63 |
| hsa-miR-191* | 33.62 | 34.20 | 35.57 | 35.15 | 34.96 | 34.99 |
| Blank (H20) |  |  |  |  |  |  |
| hsa-miR-1204 |  |  |  |  |  |  |
| hsa-miR-548j | 35.24 | 35.74 | 35.42 | 37.70 | 36.50 | 35.66 |
| hsa-miR-555 | 37.25 |  |  |  | 38.57 | 39.81 |
| hsa-miR-1224-3p | 32.34 | 32.16 | 32.20 | 32.70 | 33.00 | 32.37 |
| hsa-miR-649 |  |  | 36.55 |  |  |  |
| hsa-miR-663b | 37.45 | 37.32 | 37.63 | 37.48 | 37.17 | 37.30 |
| hsa-miR-634 |  |  |  |  |  | 37.84 |
| hsa-miR-1248 |  |  |  |  |  |  |
| hsa-miR-889 | 33.66 | 34.69 | 35.39 | 35.12 | 33.31 | 34.21 |
| hsa-miR-1227 | 35.88 | 36.16 | 35.92 | 36.50 | 37.32 | 35.90 |
| hsa-miR-517b | 37.66 |  |  |  | 38.05 | 37.32 |
| hsa-miR-1255b |  | 37.24 |  |  | 39.99 | 36.64 |
| hsa-miR-330-5p | 36.22 |  | 38.21 | 37.95 | 36.31 | 39.52 |
| hsa-miR-1238 | 36.49 | 37.34 | 36.68 |  |  | 35.44 |
| hsa-miR-188-3p | 34.61 | 33.70 | 34.67 | 35.37 | 34.44 | 34.10 |
| hsa-miR-589* | 33.73 | 33.26 | 34.26 | 32.47 | 32.66 | 34.01 |
| hsa-miR-125b-2* | 33.22 | 33.28 | 35.87 | 37.47 | 37.57 | 36.17 |
| hsa-miR-16-2* | 31.27 | 32.03 | 31.17 | 31.50 | 30.56 | 30.31 |
| hsa-miR-515-5p |  |  | 38.02 |  |  |  |
| hsa-miR-340* | 34.11 | 33.99 | 34.71 | 35.83 | 35.18 | 35.17 |
| UniSp3 IPC | 22.63 | 22.03 | 22.39 | 22.64 | 22.50 | 21.91 |
| hsa-miR-34a* | 33.83 | 34.00 | 36.59 | 35.61 | 36.11 |  |
| hsa-miR-342-5p | 34.76 | 35.01 | 35.99 | 35.40 | 36.05 | 34.04 |
| hsa-miR-639 | 34.63 | 34.13 | 34.24 | 33.90 | 35.18 | 33.73 |
| hsa-let-7i* | 31.26 | 31.56 | 32.06 | 32.50 | 30.96 | 31.13 |
| SNORD38B | 36.74 |  |  | 39.51 | 33.53 | 33.36 |
| hsa-miR-645 | 37.02 | 36.52 | 39.01 | 38.24 | 38.28 |  |
| (hsa-miR-1259) |  |  |  |  |  |  |
| hsa-miR-33a* | 33.96 | 33.90 | 34.00 | 34.64 | 35.40 | 35.25 |
| hsa-miR-609 | 36.80 | 37.21 | 37.04 | 39.29 | 39.04 | 39.79 |
| hsa-miR-34b* | 37.90 | 36.11 | 38.03 |  | 37.27 | 35.98 |
| hsa-miR-579 | 35.35 | 35.72 | 36.24 | 34.52 | 34.59 | 34.37 |
| hsa-miR-379* | 35.80 | 37.49 | 36.66 | 37.19 | 34.50 | 35.19 |
| hsa-miR-1914* | 38.79 |  | 39.39 |  |  |  |
| hsa-miR-614 |  |  |  |  | 35.61 | 38.86 |
| (hsa-miR-1974) | 25.44 | 24.77 | 25.54 | 25.49 | 25.27 | 24.54 |
| hsa-miR-93* | 31.44 | 31.73 | 31.70 | 32.33 | 31.26 | 30.80 |
| hsa-miR-219-2-3p |  |  | 39.19 |  |  | 39.65 |
| hsa-miR-616 | 36.14 | 36.08 | 39.15 | 38.01 | 39.51 | 36.71 |
| hsa-miR-1912 |  | 37.37 | 37.99 | 37.40 |  |  |
| hsa-miR-2110 | 32.88 | 33.20 | 33.49 | 33.48 | 33.27 | 32.43 |
| hsa-miR-626 |  |  |  |  |  |  |
| hsa-miR-200a* | 35.57 | 36.56 | 36.16 | 36.84 | 38.07 | 37.08 |
| hsa-miR-432* | 37.53 | 39.02 | 38.09 | 38.98 | 39.17 | 39.01 |
| hsa-miR-636 | 32.53 | 32.44 | 32.71 | 32.44 | 32.69 | 32.31 |
| hsa-miR-888* | 39.05 |  |  |  |  | 37.74 |
| hsa-miR-450b-3p | 38.83 | 35.99 | 38.85 | 37.35 | 36.67 | 37.30 |
| Blank (H20) |  |  |  |  |  |  |
| hsa-miR-520d-3p |  |  |  | 38.40 |  | 39.43 |
| hsa-miR-1265 |  |  |  |  |  |  |
| hsa-miR-1203 | 36.15 | 34.20 | 35.78 | 36.61 | 37.61 | 35.28 |
| hsa-miR-548k |  | 38.36 |  |  | 37.66 | 38.99 |
| hsa-miR-548a-5p | 36.12 | 34.66 | 39.11 | 36.59 | 36.62 | 35.28 |
| hsa-miR-1253 | 38.78 | 37.50 | 38.24 | 39.57 |  |  |
| hsa-miR-615-5p | 36.47 | 36.67 | 38.46 | 38.24 |  | 38.45 |
| hsa-miR-1236 | 37.12 | 38.45 |  | 39.27 | 37.87 | 35.27 |
| hsa-miR-1208 | 36.18 | 35.66 | 35.33 | 35.86 | 34.99 | 36.83 |
| hsa-miR-302e | 39.18 | 38.85 | 37.04 | 37.42 |  | 39.06 |
| hsa-miR-1206 | 39.29 |  |  |  |  |  |
| hsa-miR-1270 | 39.39 |  | 37.66 |  | 36.73 | 36.55 |
| hsa-miR-525-3p |  |  |  |  |  |  |
| hsa-miR-1200 |  | 38.02 |  |  | 39.85 |  |
| hsa-miR-29b-1* | 34.61 | 34.03 | 35.75 | 35.80 | 34.98 | 33.78 |
| hsa-miR-33b* | 35.86 | 35.25 | 35.45 | 36.80 | 35.74 | 37.19 |
| hsa-miR-223* | 33.00 | 33.25 | 33.96 | 33.75 | 32.64 | 32.51 |
| hsa-miR-380* |  |  |  |  |  |  |
| hsa-miR-891b |  | 38.73 |  | 39.90 |  | 39.08 |
| hsa-miR-424* | 38.60 | 37.59 | 38.19 |  | 39.42 | 38.16 |
| hsa-miR-339-3p | 31.52 | 31.86 | 31.98 | 31.89 | 31.66 | 31.14 |
| UniSp3 IPC | 23.28 | 22.32 | 22.69 | 22.42 | 23.53 | 21.84 |
| hsa-miR-647 |  |  |  |  |  |  |
| hsa-miR-183* |  |  |  |  |  |  |
| hsa-miR-92b* | 38.00 | 36.67 | 38.34 | 38.99 | 37.18 | 39.87 |
| U6 | 32.32 | 32.41 | 33.60 | 33.41 | 34.07 | 32.56 |
| hsa-miR-146a* | 34.99 | 35.05 | 34.83 | 36.50 | 34.54 | 35.00 |
| hsa-miR-1911 |  |  | 38.58 |  |  | 39.25 |
| hsa-miR-593* | 34.16 | 33.69 | 33.87 | 35.85 | 34.52 | 34.21 |
| hsa-miR-628-5p | 39.20 | 38.09 | 36.90 |  | 37.97 | 35.98 |
| hsa-miR-767-3p |  |  |  |  |  |  |
| hsa-miR-518d-3p |  | 39.07 |  |  |  |  |
| hsa-miR-24-1* | 32.52 | 32.77 | 34.89 | 35.01 | 36.65 | 37.41 |
| hsa-miR-544 | 34.47 | 34.55 | 35.78 | 35.42 | 34.50 | 34.60 |
| hsa-miR-193b* | 33.27 | 32.92 | 37.99 | 35.40 | 38.65 | 36.52 |
| hsa-miR-577 | 36.75 | 34.69 | 36.05 | 36.69 | 36.04 | 34.99 |
| hsa-miR-541* | 36.75 | 36.97 | 35.96 | 35.82 | 36.15 | 36.76 |
| hsa-miR-208a | 39.73 | 37.04 | 38.47 | 38.28 | 37.47 | 35.77 |
| hsa-miR-629* | 37.15 | 35.29 | 36.88 | 36.90 | 35.32 | 38.66 |
| hsa-miR-1207-5p | 33.51 | 33.50 | 34.26 | 34.09 | 34.38 | 33.81 |
| hsa-miR-630 | 39.28 |  | 38.45 | 38.62 |  |  |
| hsa-miR-384 |  |  |  |  |  |  |
| hsa-miR-885-3p | 33.63 | 34.28 | 35.88 | 36.48 | 37.52 | 35.08 |
| hsa-miR-675* | 32.90 | 33.11 | 35.20 | 36.92 | 37.15 | 36.16 |
| hsa-let-7g* | 33.06 | 33.63 | 35.44 | 34.87 | 33.71 | 33.99 |
| hsa-miR-526b* |  | 37.84 |  |  |  |  |
| UniSp3 IPC | 23.11 | 22.58 | 22.51 | 22.47 | 23.47 | 22.16 |
| Blank (H20) |  |  |  |  |  |  |
| hsa-miR-552 | 34.51 | 34.48 | 35.03 | 35.44 | 35.03 | 34.73 |
| hsa-miR-513c |  |  | 39.53 |  |  |  |
| hsa-miR-331-5p | 34.32 | 35.14 | 35.73 | 35.30 | 36.27 | 34.73 |
| hsa-miR-1182 |  |  |  | 39.54 |  | 39.17 |
| hsa-miR-611 | 36.63 | 39.36 |  | 38.70 | 37.65 | 37.49 |
| hsa-miR-1181 | 34.71 | 35.20 | 36.89 | 35.78 | 36.52 | 34.79 |
| hsa-miR-638 | 30.46 | 30.13 | 30.94 | 31.11 | 32.08 | 30.25 |
| hsa-miR-1258 | 37.94 | 38.69 |  |  |  | 39.90 |
| hsa-miR-650 | 36.44 | 35.41 | 36.79 | 36.59 | 35.83 | 36.00 |
| hsa-miR-1178 |  |  | 38.09 |  |  |  |
| hsa-miR-600 | 38.16 |  |  |  |  |  |
| hsa-miR-599 |  |  |  |  |  |  |
| hsa-miR-520g |  | 39.30 |  |  |  | 38.44 |
| hsa-miR-564 | 35.61 | 36.65 | 35.53 | 36.05 | 36.63 | 35.43 |
| hsa-miR-132* |  |  |  |  | 36.28 |  |
| hsa-miR-450b-5p |  |  |  |  | 37.82 |  |
| hsa-miR-485-3p | 28.16 | 27.40 | 28.18 | 28.20 | 28.23 | 28.07 |
| hsa-let-7f-2* | 31.64 | 32.08 | 32.50 | 32.69 | 33.39 | 32.58 |
| hsa-miR-155* | 37.60 |  | 38.37 | 38.95 |  |  |
| hsa-miR-105* | 32.17 | 31.51 | 32.22 | 33.06 | 32.34 | 32.21 |
| hsa-miR-486-3p | 35.18 | 35.22 | 34.87 | 34.67 | 34.36 | 33.43 |
| hsa-miR-320b | 25.36 | 25.37 | 26.20 | 26.11 | 26.07 | 25.22 |
| hsa-miR-296-3p | 37.73 | 38.95 |  |  |  |  |
| hsa-miR-7-2* | 37.51 | 39.37 | 38.54 |  |  | 38.07 |
| hsa-miR-550a* | 35.08 | 35.27 | 35.26 | 34.38 | 35.26 | 33.40 |
| SNORD49A |  | 35.72 |  |  |  |  |
| hsa-miR-593 | 36.99 | 35.47 | 35.77 | 36.32 | 36.69 | 34.49 |
| hsa-miR-605 | 29.17 | 28.19 | 28.72 | 28.86 | 28.59 | 28.79 |
| hsa-miR-493* | 34.43 | 34.20 | 34.56 | 35.60 | 32.82 | 32.04 |
| hsa-miR-1538 | 32.36 | 32.00 | 32.38 | 33.02 | 33.48 | 32.01 |
| hsa-miR-454* | 37.25 | 37.01 | 38.17 | 37.42 | 36.59 | 35.75 |
| hsa-miR-224* | 34.09 | 34.93 | 34.34 | 34.86 | 33.67 | 34.36 |
| hsa-miR-30a* | 30.61 | 31.22 | 33.15 | 33.28 | 34.50 | 32.56 |
| hsa-miR-607 |  |  |  |  |  |  |
| hsa-miR-214* | 34.13 | 36.03 | 34.61 | 35.24 | 35.76 | 36.24 |
| hsa-miR-190b | 38.34 |  |  |  | 38.61 |  |
| hsa-miR-505* | 34.23 | 34.18 | 35.24 | 36.46 | 35.75 | 33.85 |
| hsa-miR-708* | 35.48 | 35.77 | 36.53 | 37.62 | 37.14 | 35.84 |
| hsa-miRPlus-C1070 | 36.58 |  |  |  |  |  |
| hsa-miR-302a* |  | 38.85 | 39.99 | 37.50 |  | 39.94 |
| hsa-miR-130a* |  |  |  |  |  |  |
| hsa-miR-1537 | 32.43 | 33.04 | 33.39 | 32.55 | 32.54 | 32.09 |
| hsa-let-7c* | 37.94 | 36.46 | 38.03 | 39.08 |  | 34.65 |
| hsa-miR-148b* | 36.33 | 36.28 | 37.49 | 36.57 | 35.75 | 36.27 |
| hsa-miR-15a* | 33.24 | 33.89 | 34.00 | 34.10 | 34.18 | 33.08 |
| Blank (H20) |  |  |  |  |  |  |
| hsa-miR-92a-2* |  | 39.29 |  |  |  | 38.71 |
| Blank (H20) |  |  |  |  |  |  |
