# Supplementary material for: Differential Plasma MicroRNA Profiles in HBeAg Positive and HBeAg Negative Children with Chronic Hepatitis B
Source: PLoS One. 2013 Mar 4;8(3):e58236. doi: 10.1371/journal.pone.0058236 (PMC3587589; doi:10.1371/journal.pone.0058236)
Supplement: Table S2 — miRNAs identified as aberrantly expressed in the screening phase using three different normalisation strategies, reported as fold change. (DOC) [file pone.0058236.s002.doc]

Table S2, miRNAs identified as aberrantly expressed in the screening phase using three different normalisation strategies, reported as fold change.

| **Norm.** | **Global mean** | | | **U6** | | | **miR-22*, -26a, and -221** | | |
| --- | --- | --- | --- | --- | --- | --- | --- | --- | --- |
| **miRNA** | **pos v. con** | **neg v. con** | **pos v. neg** | **pos v. con** | **neg v. con** | **pos v. neg** | **pos v. con** | **neg v. con** | **pos v. neg** |
| **99a** | 51 | 10 | 5 | 43 | 5 | 9 | 57 | 13 | 5 |
| **100** | 546 | 70 | 8 | 459 | 35 | 13 | 610 | 91 | 7 |
| **122** | 161 | 27 | 6 | 135 | 14 | 10 | 180 | 35 | 5 |
| **122*** | 2233 | 332 | 7 | 1463 | 326 | 4 | 2548 | 498 | 5 |
| **125b** | 25 | 5 | 5 | 21 | 3 | 9 | 28 | 6 | 5 |
| **192** | 54 | 8 | 7 | 45 | 4 | 11 | 60 | 10 | 6 |
| **192*** | 373 | 65 | 6 | 245 | 64 | 4 | 426 | 98 | 4 |
| **193b** | 27 | 5 | 6 | 23 | 3 | 9 | 30 | 6 | 5 |
| **194** | 154 | 22 | 7 | 130 | 11 | 12 | 172 | 29 | 6 |
| **215** | 63 | 8 | 7 | 53 | 4 | 13 | 70 | 11 | 6 |
| **365** | 17 | 3 | 5 | 14 | 3 | 8 | 19 | 4 | 4 |
| **455-5p** | 34 | 7 | 5 | 29 | 3 | 9 | 38 | 9 | 4 |
| **455-3p** | 46 | 6 | 8 | 30 | 6 | 5 | 53 | 9 | 6 |
| **483-3p** | 24 | 4 | 6 | 20 | 3 | 10 | 27 | 5 | 5 |
| **885-5p** | 213 | 25 | 8 | 179 | 13 | 14 | 237 | 33 | 7 |
| **1247** | 20 | 3 | 7 | 13 | 3 | 5 | 23 | 4 | 5 |

Footnote:

Norm.: Normalisation strategy

miR-22*, -26a, and -221: Geometric mean of miR-22*, -26a, and -221

Pos v. con: HBeAg positive versus controls

Neg v. con: HBeAg negative versus controls

Pos v. neg: HBeAg positive versus HBeAg negative
