# Supplementary material for: Differential Plasma MicroRNA Profiles in HBeAg Positive and HBeAg Negative Children with Chronic Hepatitis B
Source: PLoS One. 2013 Mar 4;8(3):e58236. doi: 10.1371/journal.pone.0058236 (PMC3587589; doi:10.1371/journal.pone.0058236)
Supplement: Table S3 — Plasma levels of circulating miRNAs and varying time from collection of blood samples to centrifugation and separation. (DOC) [file pone.0058236.s003.doc]

Table S3, Plasma levels of circulating miRNAs and varying time from collection of blood samples to centrifugation and separation.

| **miRNA** | **Sample** | **Mean C_T_ (±SD)** | | **P-value** |
| --- | --- | --- | --- | --- |
|  |  | **<4 hours** | **>4 hours** |  |
| **99a** | HBeAg pos | 26.4 (1.4) | 26.0 (1.5) | 0.6 |
|  | HBeAg neg | 29.7 (1.9) | 29.4 (1.8) | 0.3 |
| **100** | HBeAg pos | 31.0 (2.2) | 30.0 (2.1) | 0.6 |
|  | HBeAg neg | 34.1 (2.0) | 33.7 (2.3) | 0.3 |
| **122** | HBeAg pos | 22.2 (1.7) | 21.4 (1.5) | 0.8 |
|  | HBeAg neg | 25.4 (1.5) | 25.2 (2.5) | 0.5 |
| **122*** | HBeAg pos | 28.6 (1.9) | 28.0 (1.7) | 0.8 |
|  | HBeAg neg | 32.3 (3.2) | 31.9 (2.8) | 0.5 |
| **125b** | HBeAg pos | 27.1 (1.6) | 26.6 (1.4) | 0.7 |
|  | HBeAg neg | 29.8 (1.5) | 30.0 (1.7) | 0.6 |
| **192** | HBeAg pos | 25.3 (1.7) | 25.0 (1.3) | 0.4 |
|  | HBeAg neg | 28.3 (1.2) | 28.2 (1.7) | 0.3 |
| **192*** | HBeAg pos | 31.9 (1.5) | 31.6 (1.5) | 0.5 |
|  | HBeAg neg | 35.1 (1.4) | 35.1 (2.2) | 0.4 |
| **193b** | HBeAg pos | 29.2 (1.8) | 28.6 (1.3) | 0.8 |
|  | HBeAg neg | 31.9 (1.7) | 32.0 (1.6) | 0.7 |
| **194** | HBeAg pos | 26.9 (2.0) | 26.3 (1.7) | 0.7 |
|  | HBeAg neg | 29.7 (1.5) | 30.0 (1.2) | 0.5 |
| **215** | HBeAg pos | 26.8 (1.9) | 26.1 (1.5) | 0.5 |
|  | HBeAg neg | 29.7 (1.8) | 29.8 (1.3) | 0.2 |
| **365** | HBeAg pos | 29.1 (1.8) | 28.5 (1.5) | 0.6 |
|  | HBeAg neg | 31.3 (1.1) | 31.7 (1.6) | 0.5 |
| **455-5p** | HBeAg pos | 32.5 (1.8) | 31.8 (1.8) | 0.4 |
|  | HBeAg neg | 35.6 (2.1) | 35.8 (1.7) | 0.5 |
| **455-3p** | HBeAg pos | 28.6 (1.6) | 27.9 (1.8) | 0.4 |
|  | HBeAg neg | 30.5 (1.9) | 32.0 (2.3) | 0.9 |
| **483-3p** | HBeAg pos | 32.8 (2.4) | 31.8 (1.7) | 0.4 |
|  | HBeAg neg | 34.6 (1.9) | 35.5 (1.8) | 0.9 |
| **885-5p** | HBeAg pos | 29.9 (1.9) | 29.0 (1.4) | 0.3 |
|  | HBeAg neg | 32.8 (2.1) | 33.1 (1.9) | 0.4 |
| **1247** | HBeAg pos | 34.7 (2.8) | 34.1 (2.1) | 0.8 |
|  | HBeAg neg | 37.5 (2.2) | 37.4 (1.5) | 0.2 |

Footnote:

The time from collection of blood samples to centrifugation and separation varied from less than 4 hours to a maximum of 48 hours. We compared the plasma levels of 16 miRNAs in 25 samples (16 HBeAg positive and 9 HBeAg negative) processed in less than 4 hours and in 35 samples (18 HBeAg positive and 17 HBeAg negative) processed after a delay of up to 48 hours.
